# (5′*S*) 5′,8-cyclo-2′-deoxyadenosine Cannot Stop BER. Clustered DNA Lesion Studies

**DOI:** 10.3390/ijms22115934

**Published:** 2021-05-31

**Authors:** Boleslaw T. Karwowski

**Affiliations:** DNA Damage Laboratory of Food Science Department, Faculty of Pharmacy, Medical University of Lodz, ul. Muszynskiego 1, 90-151 Lodz, Poland; Boleslaw.Karwowski@umed.lodz.pl

**Keywords:** 5′,8-cyclo-2′-deoxyadenosine, DNA damage, DNA repair, Polβ, Okazaki-like fragments, xrs5

## Abstract

As a result of external and endocellular physical-chemical factors, every day approximately ~10^5^ DNA lesions might be formed in each human cell. During evolution, living organisms have developed numerous repair systems, of which Base Excision Repair (BER) is the most common. 5′,8-cyclo-2′-deoxyadenosine (cdA) is a tandem lesion that is removed by the Nucleotide Excision Repair (NER) mechanism. Previously, it was assumed that BER machinery was not able to remove (5′*S*)cdA from the genome. In this study; however, it has been demonstrated that, if (5′*S*)cdA is a part of a single-stranded clustered DNA lesion, it can be removed from *ds*-DNA by BER. The above is theoretically possible in two cases: (A) When, during repair, clustered lesions form Okazaki-like fragments; or (B) when the (5′*S*)cdA moiety is located in the oligonucleotide strand on the 3′-end side of the adjacent DNA damage site, but not when it appears at the opposite 5′-end side. To explain this phenomenon, pure enzymes involved in BER were used (polymerase β (Polβ), a Proliferating Cell Nuclear Antigen (PCNA), and the X-Ray Repair Cross-Complementing Protein 1 (XRCC1)), as well as the Nuclear Extract (NE) from xrs5 cells. It has been found that Polβ can effectively elongate the primer strand in the presence of XRCC1 or PCNA. Moreover, supplementation of the NE from xrs5 cells with Polβ (artificial Polβ overexpression) forced oligonucleotide repair via BER in all the discussed cases.

## 1. Introduction

DNA is a miraculously fragile molecule in which the secret code of life can be found. The double helix, formed by two complementary strands which contain four nucleotides (nucleotide unit; i.e., nucleoside connected to a phosphodiester bond) is continuously exposed to the activity of harmful endo- and exocellular physical-chemical factors [1]. Reactive Oxygen Species (ROS) pose a threat to the stability and integrity of genetic information. The presence of ROS next to the double helix can lead to a wide range of lesions. Up to now, around 80 different types of DNA damage have been identified [2,3]. As shown in Figure S1, four main types of nucleic acid lesions can be listed with regard to the lesion distribution criteria, that is, **(1)** isolated lesions—one lesion per one or two helix turns, **(2)** tandem lesions—a particular type of clustered DNA damage consisting of two contiguously damaged nucleosides or two adjacent modifications (e.g., in the sugar and base moieties), resulting from a single free radical initiating event [4], **(3)** single-stranded clustered DNA lesions—two or more DNA lesions per one or two double helix turns located in the same *ds*-DNA strand, **(4)** double-stranded clustered DNA lesions—two or more DNA lesions per one or two double helix turns located in both *ds*-DNA strands: matrix and complementary strand.

It is estimated that every hour 3 × 10^17^ DNA lesions are being produced in the human body [5]. The most common are the Apurinic/Apyrimidinic site (AP-site), Single-Strand Break (SSB), 7,8-dihydro-8-oxo-2′-deoxyguanosine (^oxo^dG), and 2′-deoxyuridine (dU) [2]. On the other hand, the frequency of Double-Strand Breaks (DSBs) is estimated only as ~50 DSB per cell cycle [1,6]. The situation becomes different with the introduction of external radiation and chemical factors to the cellular environment. Ward et al. proposed that the differences in lethality depend on the spatial lesions’ distribution [7]. However, the effectiveness of DNA damage repair depends also on the nature of the damage. Take, for example, the Interstrand Cross-Links (ICLs) [8]. It has been assumed that the appearance of 40 ICLs in the bacterial genome is lethal [9]. The appearance of ICLs in cellular DNA is a result of the chemical agents (such as psoralen, mitomycin, cis-/transplatin) interaction with the double helix [8]. Moreover, other anticancer drugs (such as doxorubicin and tirapazamine) can cause hard-to-remove DNA damage [10]. Local Multiple Damage Sites (LMDSs) appearing in the genome become much more problematic for cell repair machinery. This thesis is well supported by the fact that single/isolated DNA lesions produced endogenously in a cell are well repaired and non-lethal. However, the presence of such damage in Clustered DNA Lesion (CDL) structures renders them harmful and mutagenic. It is estimated that a radiation dose of 1Gy can generate 1000 SSBs, 2000 nucleobase damage events (e.g., oxidation), 250 AP-sites, 40 DSBs, and 122 non-DSB LMDSs in a cell [11]. Approximately 20% of CDLs contain up to three lesions [12,13,14]. For more details, please see the reviews written by Sage [15,16]. During any discussion about CDL formation, the frequency of elementary DNA damage and its source cannot be ignored [17,18,19]. It should be pointed out the majority of single lesions (e.g., AP-site, dU, ^oxo^dG, etc.) are repaired by Base Excision Repair (BER) machinery, which is the main system of their removal and repair (Figure S2) [20,21]. However, the appearance of two or more DNA lesions in the vicinity harms the fidelity and agility of the repair processes; their presence can result in mutation induction or chromosomal aberrations [22]. O’Neill, Wallace, and Weinfeld proved that the repair activity of cellular extracts and BER enzymes decreased when a plasmid contained clustered DNA lesions with damage other than DSBs [23,24,25,26].

The presence of a 5′,8-cyclo-2′-deoxyadenosine (cdA) in the genome makes the repair processes more difficult. The (5′*R*)cdA and (5′*S*)cdA are removed 40 and 150 times slower, respectively, than *cis*-platin-DNA adducts [27]. CdA belongs to the tandem DNA lesion type. As shown in Figure S1, in the structure of the discussed nucleoside, after 5′,8 endomolecular cyclization initiated exclusively by •OH activity (5′ or 5″ proton abstraction), both moieties of the nucleoside (i.e., 2-deoxyribose and the nucleobase) were damaged [28]. Noteworthy, cdPus are removed from oligonucleosides by the Nucleotide Excision Repair (NER) system, not by BER, as no cdPu-specific glycosylases are known. The defect in the cellular NER system may lead to an accumulation of DNA damage sites, which causes various clinical manifestations (e.g., *Xeroderma Pigmentosum*, TrichoThioDystrophy, and Cockayne syndrome) [29,30]. Additionally, the 5′*R* and 5′*S* diastereomers of cdA strongly influence dU and AP-site incision by uracil-DNA glycosylase (UDG) and human AP-site endonuclease one (hAPE1). The incision rate also depends on the mutual distribution of the lesions in the same DNA strand and on the chirality of cdA C5′ carbon [31,32,33,34].

Moreover, any cdA diastereomer present in the double helix structure has a negative impact on the activity of different polymerases (T7 DNA Polymerase, Polδ, Polη) [27,35,36]. The (5′*S*)cdA also induces the loop formation in the matrix strand which consists of trinucleotide repetitions CGA. This creates a long flap that is skipped over by DNA Polβ (lesion bypass synthesis) which subsequently may lead to repeated deletions and genomic instability [37,38]. Previous studies have shown that the presence of (5′*S*)cdA exerts a pronounced influence on the 3D structure of *ds*-DNA [39]. These results also indicate that the (5′*S*)cdA located on the 5′-end side of the repaired lesion, in the complementary strand, is the critical inhibiting factor of the BER process [39].

This article has shown the possibility that, if (5′*S*)cdA (as an example of 5′,8-cyclo-2′-deoxyPurines) becomes a part of a single-stranded clustered DNA lesion, it can be successfully removed from the genome by proteins involved in BER machinery.

## 2. Results and Discussion

### 2.1. Hypothesis: How and When BER Proteins Remove (5′S)cdA from the Genome?

**Figure 1:**
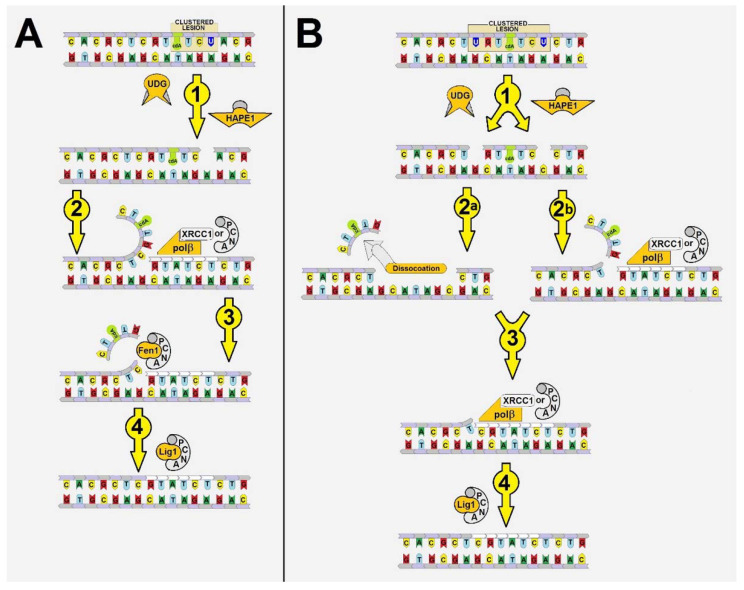
Graphical representation of two possible mechanisms of (5′*S*)cdA as a part of clustered lesion removal from the genome. (**A**) Flipping out the descending part of the oligonucleotide with subsequent FEN1 action (1—SSB formation by glycosylase and endonuclease; 2—polymerase action solely or as a complex with PCNA or XRCC1; 3—a flipped-out oligonucleotide as a result of FEN1 activity; 4—strand reconstitution by Lig1). (**B**) Removal of a short, single-stranded oligonucleotide fragment containing (5′*S*)cdA under a thermodynamic regime (1—formation of two SSBs by glycosylase and endonuclease; 2a—spontaneous dissociation of a short, single-stranded oligomer; or 2b—dissociation of a single-stranded oligomer flipped-out by the polymerase activity (solely or as a complex with another protein); 3—“filling out” the missing part of the elongated strand; 4—strand ligation by Lig1). UDG (uracil-DNA glycosylase); hAPE1 (human AP-site endonuclease one); Polβ (polymerase β); XRCC1 (X-ray repair cross-complementing protein 1); PCNA (proliferating cell nuclear antigen); FEN1 (flap structure-specific endonuclease 1); Lig1 (Ligase one); SSB (single strand break).

As shown in Figure 1A,B, there are two possibilities for (5′*S*)cdA removal from the genome by BER proteins. Both are hypothetically “allowed” yet never taken under consideration.

**(1)** First possibility

Figure 1A represents the situation where lesions were induced on the 5′-end side defined by the (5′*S*)cdA. If DNA damage is the suitable substrate for specific glycosylases, subsequently formed SSBs could be filled in by a polymerization complex (e.g., PCNA-Polδ/ε or Polβ)**.** Therefore, if BER proteins act upon the double helix “behind” the (5′*S*)cdA site (towards its 5′-end), then the genome will be liberated from this lesion.

**(2)** Second possibility

The processes depicted in Figure 1B are based on the idea of Okazaki-like fragment formation [40,41]. (5′*S*)cdA is located between two lesions (in the same strand) which, after glycosylase and/or endonuclease action, become two SSBs surrounding (5′*S*)cdA. This situation brings about two results: **(A)** The short oligomer containing (5′*S*)cdA can spontaneously dissociate from the double helix; **(B)** the polymerase (solely or in cooperation with PCNA or XRCC1) will “unpick” the descending oligomer containing (5′*S*)cdA during the primer elongation step, until it becomes detached from the complementary/matrix strand.

**Figure 2:**
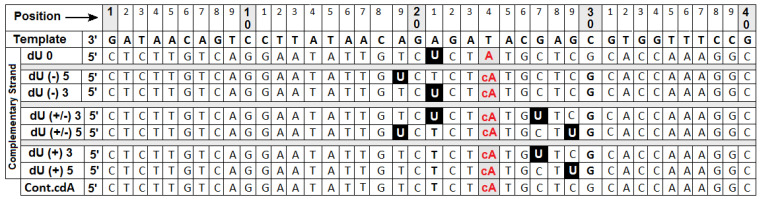
Sequence of oligonucleotides containing 2′-deoxyUridine (U) in black square and (5′*S*) 5′,8-cyclo-2′-deoxyadenosine (cA) in red color. After UDG and hAPE1 digestion, the U was converted to a Single-Strand Break (SSB) but the *ds*-oligo notation was unchanged.

To test this hypothesis, a set of experiments was performed using *ds*-oligonucleotides (sequences are presented in Figure 2) with subsequent dU to SSB conversion.

The dU was chosen as a model DNA lesion due to efficient glycosidic bond hydrolysis by UGD to the AP-site and subsequent conversion to the SSB by hAPE1. Noteworthy, dU has an established application for DNA repair studies with various cellular extracts [26,39,42,43]. Moreover, dU frequently appears in the genome as a product of dC deamination (~500 per day per cell or ~10^4^ per genome per cell), or dUTP misincorporation [44]. The *ds*-DNA denoted as dU0 contains only one dU or SSB as a lesion and was used as a control oligonucleotide throughout experiments presented below.

The following system was used to notate the mutual positions of DNA lesions: the location of dU or SSB in the DNA strand on the 5′-OH site of the (5′*S*)cdA was denoted as negative (-) and on the 3′-OH as positive (+). Synthesized single-stranded 40-mers were verified on the 20% PAGE with 8M urea after ^32^P-labeling on the 5′-end. The purity of the prepared duplexes was checked on the non-denaturing 20% PAGE (see Figure S3). For all investigated *ds*-oligonucleotides, melting temperature (Tm) was found to be higher than 75 °C. The stability of an oligonucleotide containing only (5′*S*)cdA as a “single” DNA lesion (denoted as Cont.cdA) was checked in the presence of glycosylases (formamidopyrimidine glycosylase (FPG), endonuclease III (Nth), UDG), NE, and hot 1M piperidine (Figure S3B and description *ibid.*). The results revealed that the Cont.cdA was not a substrate for either of the above enzymes, nor NE, and was stable in the discussed experimental conditions. Moreover, Cont.cdA was not digested by hot 1M piperidine, as expected. The above observation confirms results from a previous study [39]. 

Moreover, in the recent article by Boguszewska et al. it was shown that, for bi-stranded CDL, when the distance between (5′*S*)cdA and AP-site is bigger than seven nucleotides, the activity of AP-site repair via the BER system was unchanged [45]. Additionally, this was also noted for (5′*R*)cdA and both diastereomeric forms of 5′,8-cyclo-2′-deoxyguanosine (5′*S* and 5′*R*). The above is in good agreement with the work of Karwowski et al. [39]. It showed that (5′*S*)cdA present in the complementary strand strongly affects DNA repair on a distance shorter than eight nucleotides between (5′*S*)cdA and AP-site in both 3′-end and 5′-end directions. However, future studies of the single- and bi-stranded CDLs, in which the distances between the discussed lesions are extended continuously from two to seven nucleotides, are definitely needed.

**Figure 3:**
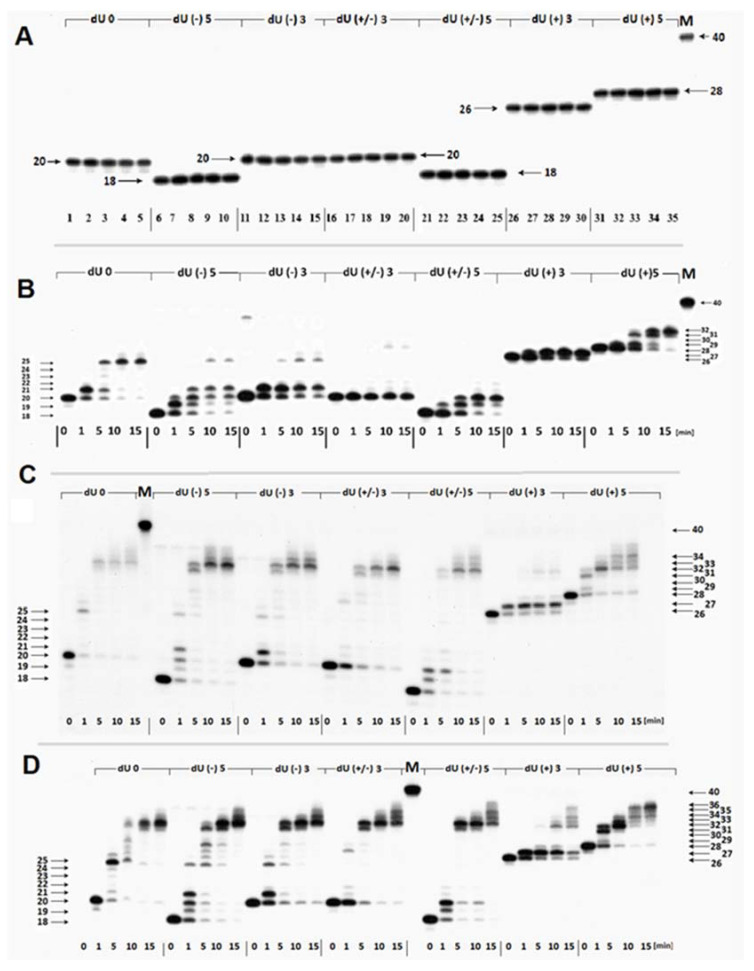
(**A**) The stability of the oligonucleotides (Figure 2) (3.63 pmol) after 30 min incubation at 37 °C: Oligonucleotides dissolved in water (lanes)—1, 6, 11, 16, 21, 26, 31; oligonucleotides dissolved in reaction buffer—2, 7, 12, 17, 22, 27, 32; oligonucleotides dissolved in reaction buffer without dNTP in the presence of Polβ (0.26 pmol)—3, 8, 13, 18, 23, 28, 33; oligonucleotides dissolved in reaction buffer without dNTP in the presence of PCNA (0.32 pmol)—4, 9, 14, 19, 24, 34; oligonucleotides dissolved in reaction buffer without dNTP in the presence of XRCC1 (0.21 pmol)—5, 10, 15, 20, 25, 35; (**B**) the influence of clustered DNA lesion on Polβ (0.26 pmol) strand-displacement in the presence of LMDS; (**C**) the influence of XRCC1 (0.21 pmol) on strand elongation by Polβ (0.26 pmol) in the presence of LMDS; (**D**) the influence of PCNA (0.32 pmol) on strand elongation by Polβ (0.26 pmol) in the presence of LMDS. (All reactions (**B**–**D**) were carried out in the repair buffer (8 μL) at 37 °C. The numbers in the vertical position indicate the oligonucleotide length. The sequences of *ds*-oligos are presented in Figure 2). M (the 40-mer oligonucleotide, the length marker, dNTP (2′-deoxynucleosides triphosphate); Polβ (polymerase β); XRCC1 (X-ray repair cross-complementing protein 1); PCNA (proliferating cell nuclear antigen); LMDS (Local Multiple Damage Sites).

To test the first hypothesis (Figure 1A), the following enzymes were selected solely or in combination: Polβ, XRCC1, PCNA, Lig1, Lig3. In the initial step of the experiment sets, the dUs were converted to suitable SSBs by UDG and hAPE1 treatment. As shown in Figure 3A, the precipitated *ds*-oligos are stable in the presence of PCNA, XRCC1, Polβ (without dNTP, to avoid polymerase action).

### 2.2. The Influence of the (5′S)cdA on Polβ Activity 

**Figure 4:**
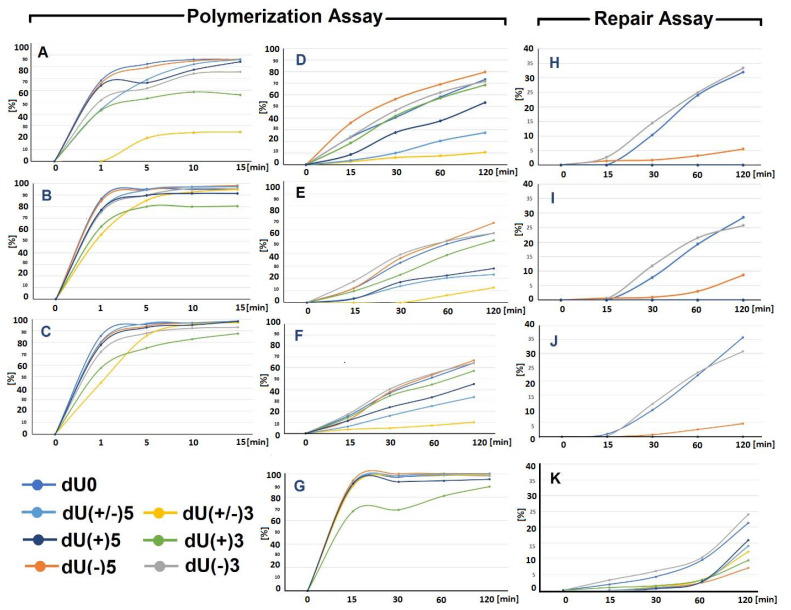
The timescale of the primer or descending strand elongation containing clustered DNA lesion hybridized to the non-damaged template strand, after UDG and hAPE1 digestion by: (**A**) Polβ, (**B**) Polβ/XRCC1, (**C**) Polβ/PCNA, (**D**) nuclear extract of xrs5 cells (NE), (**E**) NE/PCNA from an external source, (**F**) NE/XRCC1 from an external source, (**G**) NE/Polβ from an external source. The repair assays of oligonucleotides after UDG and hAPE1 digestion by: (**H**) NE, (**I**) NE/PCNA from an external source, (**J**) NE/XRCC1 from an external source, (**K**) NE/Polβ from an external source. The numeric raw data presented in the graphs, the average, standard deviation values and raw radiograms are given in a separate file in the Supplementary Materials as Table S1 and Figures S6 and S7. The sequences of single-stranded 40-mer oligonucleotides are given in Figure 2. UDG (uracil-DNA glycosylase); hAPE1 (human AP-site endonuclease one); Polβ (polymerase β); XRCC1 (X-ray repair cross-complementing protein 1); PCNA (proliferating cell nuclear antigen); NE (nuclear extract from xrs5 cells).

Synthesized oligonucleotides after hybridization with a complementary strand (Figure 2) were converted to suitable *ds*-oligonucleotides with a SSB (as a result of reaction with UDG and hAPE1). The obtained *ds*-oligonucleotides with SSB were used to determine the influence of (5′S)cdA on Polβ activity. As depicted in Figure 3B, Polβ can elongate the primer strand in a duplex with a SSB (control) (i.e., dU0 up to five nucleotides). This observation is in good agreement with previous data postulated and presented by Hoffmann et al. [46]. Surprisingly, different *ds*-oligos were elongated differently (different number of nucleotide units inserted by Polβ): dU(−)5 up to seven nucleotides, dU(−3) up to five, dU(+/−)3 up to seven, dU(+/−)5 up to four, dU(+)3 up to on, and dU(+5) up to four nucleotides. However, for the investigated *ds*-oligos, the main products of the reaction had different length: For dU0 it was 25-mer; for dU(−5) it was 20/21-mer; for dU(−)3 it was 21-mer; for dU(+/−)3 it was 20-mer (no polymerization was observed); for dU(+/−)5 it was 20-mer, for dU(+)3 it was 27-mer; and for dU(+5) it was 32-mer. Densitometry analysis revealed the following order of the total elongation efficiency, catalyzed by Polβ: dU0 > dU(−)5 > dU(+/−)5 > dU(+)5 > dU(−)3 > dU(+)3 > dU(+/−)3 (Figure 4A, Table S1A).

Based on these results, it can be predicted that mutual (5′*S*)cdA/dU spatial distribution is crucial for Polβ action; as the distance between lesions increases, the elongation process becomes more effective. However, the Polβ activity was less impaired when the SSB was located on the 5′-end of (5′*S*)cdA than on the 3′-end. Additionally, Polβ elongated a primer strand by five and seven nucleotides (dU(−)3 and dU(−)5, respectively). Therefore, it can be postulated that in both cases, the flipped out oligomer (a potential substrate for flap structure-specific endonuclease 1 (FEN1)) contained (5′*S*)cdA. Interestingly, it was observed that Polβ productivity was the weakest (almost negligible) for “pro-gapped” dU(+/−)3. It indicates that the small *ss*-oligo fragment d[CT((5′*S*)cdA)TG] detached from the double helix, leaving a broad gap in its structure, which was similar to an Okazaki fragment. This has been supported by the theoretically predicted Tm values of a short double-stranded duplex (d[CTATG] * d[CATAG]), which was 16 °C [47]. At this point, it should be noted that (5′*S*)cdA destabilized the double helix structure and; therefore, decreased Tm by ~6 °C. A different effect was observed for dU(+/−)5, in which the distance between two SSBs was extended to nine nucleotides. However, Polβ stopped elongation after inserting two nucleotides, instead of seven or a minimum of three, as found for dU(−)5 (Figure 3B). The predicted Tm for d[CTCTATGCT]*d[AGCATAGAG] was approximately 10 °C higher than for a native pentamer (i.e., 26 °C). Therefore, when Polβ started to elongate the primer strand (by more than one nucleotide), nucleotide units of the descending strand were flipped out one by one. This may have decreased the stability of the discussed *ds*-DNA fragment up to a point of spontaneous gap creation (Figure 1). This situation can be accelerated by the presence of (5′*S*)cdA in the discussed oligo fragment. Hence, the gap formation was privileged in the case of dU(+/−)3 and dU(+/−)5. For these constructs, negligible and limited Polβ activity was observed, respectively, as shown in Figure 3B. Gapped *ds*-DNA was not a suitable substrate for the polymerase β. This was in good agreement with previous data, which indicated that Polβ alone is not involved in lagging strand maturation [40]. 

From a mechanistic and structural point of view, it is important to mention that in the case of dU(−)5 and dU(+)5, during strand elongation, (5′*S*)cdA was located on the outer surface of Polβ, as presented in Figure S4 [48,49]. Conversely, for dU(−)3 and dU(+)3, (5′*S*)cdA interacted with the protein’s surface (dU(+)3 near the catalytic site and dU(−)3 near the lytic site. However, in the case of dU(−)3, the downstream oligo part formed a somewhat rigid duplex structure, while in the case of dU(+/−)3 a flexible single strand was present between the lytic part and the “thumb” of the discussed polymerase. Future theoretical studies are required to elucidate this phenomenon. Moreover, it is problematic for small proteins like Polβ or PCNA to keep the template strand in the correct position of the catalytic unit for a subsequent elongation; in the case of dU(+/−)3, the only negligible activity of Polβ was noted (Figure 3B). The four nucleotide elongation of the pro-gapped parts of oligo (dU(+/−)5) up to ninemer leads to increased thermal stability and allows Polβ to add two dNMP units to the primer strand (Figure 3B).

XRCC1, a scaffold protein that plays an important role in short patch BER (SP-BER), coordinates the “binding and activity” of other repair enzymes [50,51,52]. The high binding affinity between the N-terminal dominant of XRCC1 and the “thumb” of Polβ has been shown by Kubota et al. [53], and the expected XRCC1-Polβ complex is depicted in Figure S4. As a next step, XRCC1 and Polβ cooperation was investigated with regard to clustered lesion repair.

As shown in Figure 3C, scaffold protein changed the pattern of polymerase activity. First of all, the effectiveness of Polβ/XRCC1 after 1 min was at the same level as for Polβ after 5 min. Increasing reaction time up to 15 min led to an elongation of 12 nucleotides in the case of dU0, dU(−)3, and dU(+/−)3; 14 nucleotides for dU(−)5, and dU(+/−)5; six nucleotides for dU(+)3; and eight nucleosides for dU(+)5. A densitometric analysis revealed the following order of polymerization efficiency: dU0 > dU(−)5 > dU(+/−)5 > dU(+)5 > dU(−)3 > dU(+/−)3 > dU(+)3 (Figure 4B). 

The following two observations are of particular interest:

- Firstly, the cooperation between XRCC1 and Polβ leads to an extended elongation of the primer strand. Depending on the relative (5′*S*)cdA and SSBs positions, up to 12 to 14 nucleotides may be inserted, which is commonly accepted for long patch BER (LP-BER) (in the case of control duplex (dU0) 12 nucleotide units were added). Under the same experimental conditions, Polβ solely added a maximum of seven nucleotides. These observations indicate that the descending strand containing (5′*S*)cdA was flipped out of the double helix and became a suitable substrate for further FEN1 action (Figure 1A—step 3).
- Secondly, a significant elongation of the primer strand was observed for dU(+/−)3, which was not prone to elongation by Polβ, due to its Okazaki-like fragment nature.

Hence, it can be postulated that the interaction of XRCC1 with the thumb unit of Polβ stabilized the template DNA strand, which makes the polymerization possible for the widely gapped double helix. Moreover, these results point to and confirm the previous observations (Figure 3B). The first step of the proposed mechanism is initiated by the specific glycosylase (UDG) and endonuclease (hAPE1) and leads to the removal of the short oligomer (containing (5′*S*)cdA) from the genome under a thermodynamic regime (Figure 1B—step 1). Similar results were obtained for dU(+/−)3 and dU(+/−)5. However, in the case of dU(+/−)5, the gap could be formed only after the addition of two nucleotides to a primer strand. During the insertion of two nucleotides, in the short, pro-gapped oligo fragment, the number of nucleobases bound to the double helix was reduced from nine to seven (Figure 1—path 2b) allowing the dissociation of the short oligo fragment. Therefore, the significant role of XRCC1 on Polβ activity was observed (Figure 3). This is in good agreement with the data proposed by Hoffmann et al. [46]. They postulated that XRCC1 and Polβ are involved in the lagging strand maturation process by filling the formed gap [46].

On the above scientific ground, the influence of PCNA on Polβ activity was then considered [54,55,56,57]. Since PCNA is the ring protein that clamps the double helix, the following proteins’ action may be facilitated. It should be particularly noticeable when a lesion with significant potential for structural “destruction” appears near the repaired site. The Polβ/PCNA joint action led to similar strand elongation to that obtained for Polβ/XRCC1 in the case of dU0, dU(−)5, and dU(−)3 (Figure 3D). For dU(+/−)3, dU(+/−)5, which are the precursors of the Okazaki-like fragments and may mimic them (Figure 1B—steps 2a and 2b), the extended strand was elongated by two dNMPs with higher efficiency than in the Polβ/XRCC1 experiment. As expected, PCNA exerted greater influence on Polβ activity in the case of dU(+)3 and dU(+)5, in which (5′*S*)cdA is present on the 5′ side of a SSB. It can be postulated that, in these cases, PCNA clamped the *ds*-oligo and facilitated polymerase activity. In the case of Polβ/PCNA, 2 additional nucleotide units were inserted for dU(+/−)3 and dU(+/−)5 in comparison to the Polβ/XRCC1 experiment (Figure 3C,D). A densitometric analysis revealed the following order of polymerase productivity: dU0 > dU(−)5 > dU(+/−)5 > dU(+)5 > dU(−)3 > dU(+/−)3 > dU(+)3, which was at the same level as in the previous experiment (Figure 4C). These results indicated the possibility of BER participation in the removal of (5′*S*)cdA from the genome either when cdPu becomes part of the single-stranded CDL (Figure 1A) or via the “Okazaki-like fragment” mechanism (Figure 1B). It should be pointed out that no strand ligation was found in the presence of Lig1 or Lig3 regardless of the investigated *ds*-oligo model/experiments (Figure S5A–C). It can be assumed that some part of the descending oligo (containing (5′*S*)cdA) was flipped out during polymerization, which prevented strand reconstitution. Furthermore, it may indicate the predominant tendency of the discussed *ds*-DNA with clustered damage to drive the BER process via the long patch path.

### 2.3. The Repair of Single-Stranded Clustered DNA Lesions by the xrs5 Nuclear Extract

(5′*S*) 5′,8-cyclo-2′-deoxyadenosine forces structural changes in the spatial geometry of the DNA duplex in the 3′-end and 5′-end directions of both strands [58,59]. As a result, enzymes (such as UDG and hAPE1) are not necessarily able to adjust the active site conformation to the rigid structure of (5′*S*)cdA, especially when it is too close to the repaired lesion [31,60]. Moreover, enzymatic activity depends on the chirality of C5′ of cdA. As mentioned above, LMDS in DNA pose a severe threat to the BER machinery. To date, single-stranded CDL containing (5′*S*)cdA have not been analyzed in regard to a nuclear extract of xrs5 cells. In this cell line, the Non-Homologous End Joining (NHEJ) system is inactive due to Ku80 protein dysfunction. Moreover, the NE derived from cells lacking Ku80 is unable to repair linear DNA via NER, due to the incomplete Ku/DNA-PKcs interaction [31,61]. Additionally, the lack of the above protein allows avoiding the interfering activity of Ku80 (binding to linear DNA termini or SSBs), which is crucial for the presented experiments [62,63].

The previous study considered only bi-stranded CDLs with (5′*S*)cdA and an AP-site at different interlesion separations [39]. Taking into account this study along with the influence of (5′*S*)cdA on the efficiency of isolated BER proteins, testing the hypothesis concerning a full set of BER proteins present in NE was justified (Figure 1A,B).

**Figure 5:**
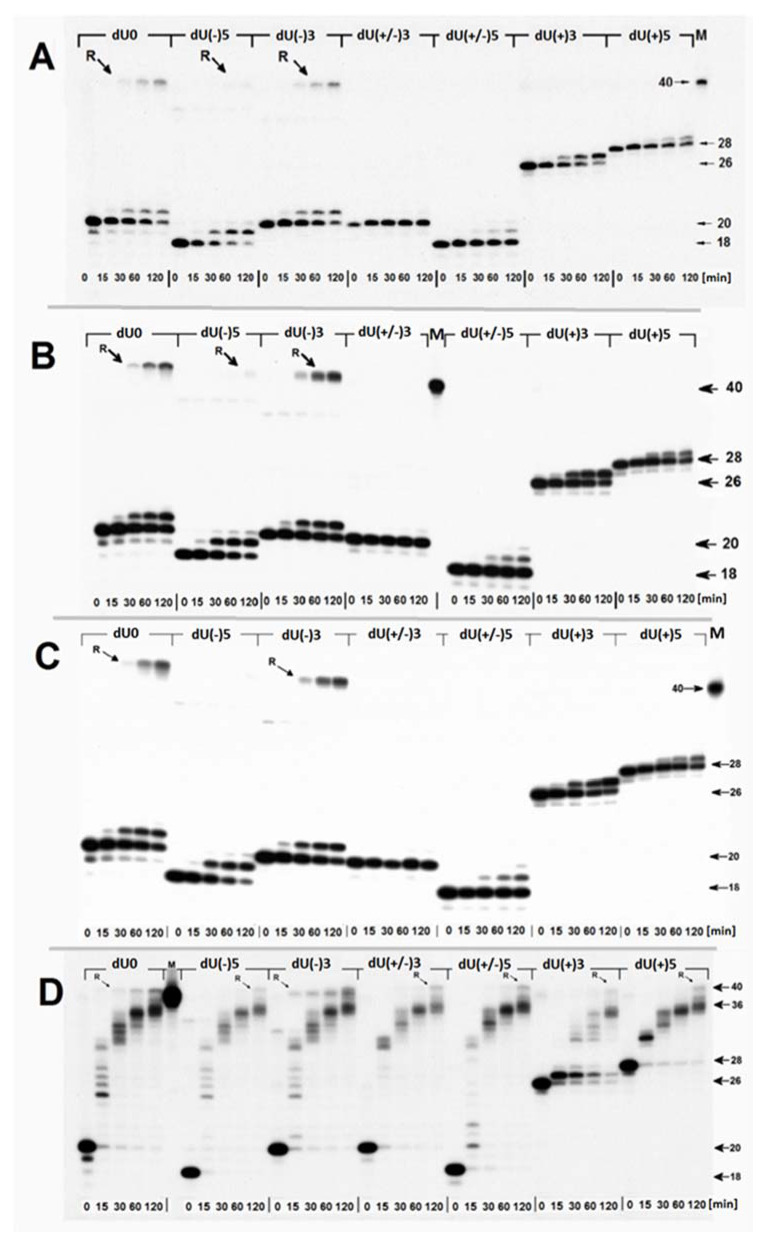
Representative denaturing polyacrylamide gels showing the rejoining (R) of an SSB, formed from the *ds*-oligonucleotides presented in Figure 2, after UDG and hAPE1 digestion by: (**A**) Nuclear extract of xrs5 cells (NE); (**B**) nuclear extract of xrs5 cells in the presence of PCNA from an external source; (**C**) nuclear extract of xrs5 cells in the presence of XRCC1 from an external source; (**D**) nuclear extract of xrs5 cells in the presence of Polβ from an external source. R (the reconstituted strand); M (the 40-mer oligonucleotide, the length marker); UDG (uracil-DNA glycosylase); hAPE1 (human AP-site endonuclease one); Polβ (polymerase β); XRCC1 (X-ray repair cross-complementing protein 1); PCNA (proliferating cell nuclear antigen); NE (nuclear extract from xrs5 cells). The numbers placed vertically indicate the oligonucleotide length.

As shown in Figure 5A, the distinct strand reconstitution was observed for the dU0 (control *ds*-oligo, which contains only a SSB) and for dU(−)3, where the SSB was located two nucleotides away in the 5′-end direction from (5′*S*)cdA. The BER effectiveness rate was noted at 30%. In the case of dU(−)5, strand rejoining was hardly visible and questionable (<5%), probably due to SSB/cdPu distance extension up to four nucleotides. For the other investigated *ds*-oligos, no repair was observed. These results indicate that (5′*S*)cdA forces the depression of BER in its 3′-end direction (dU(+)3 and dU(+)5). The same trend was observed for pro-gapped constructs (dU(+/−)3 and dU(+/−)5). An analysis of the polymerization process revealed the following order of effectiveness: dU(−)5 > dU(−3) ~ dU0 > dU(+)3 > dU(+)5 > dU(+/−)5 > dU(+/−)3. It shows that the polymerization process is different than in the case of using pure Polβ, especially for dU(+)3, dU(+)5, and dU(+/−)5 (Figure 3B and Figure 5A).

The differences observed between dU(−5) and dU(−3) can be derived from the fact that the interaction between the double helix and proteins is a three-dimensional process, dependent on the structural changes forced by (5′*S*)cdA. In a nutshell, when the polymerization or ligation occurs, the *ds*-DNA slips with rotation into the enzyme structure, rather than sliding in a 2D manner. As a result, in the case of dU(−5), (5′*S*)cdA may directly interact with proteins’ amino acids, forcing a disruption of the enzyme activity (allosteric effect), or changing the protein interaction with the descending oligomer, which was not observed for dU(−3). In the case of enzymes that have an “open” structure during oligonucleotide screwing into them (e.g., Polβ), in some cases (5′*S*)cdA occurs outside the protein, while in others it interacts directly with the active site of the protein. Therefore, dU(−)5, while under repair, could not be properly reached by the active protein site, and the BER machinery has been slowed down or stopped. However, other proteins might have been involved in this process, or the level of Polβ in the NE was insufficient. It should be pointed out that further theoretical studies (Molecular Dynamics) are vital for explaining this phenomenon in detail.

It can be postulated that other proteins are involved in this process, or the level of Polβ in the NE from xrs5 cells is different (i.e., not sufficient). As demonstrated previously, Polβ can cooperate with two scaffold proteins XRCC1 and PCNA. Therefore, to support the repair and polymerization process, the same amounts of XRCC1 and PCNA were added, as previously, to the NE (Figure 5C,D) to support the repair and polymerization process. 

Nevertheless, no significant differences were observed compared to experiments with sole NE treatment (Figure 4A,B,I,J). The yield of strand reconstitution adopted an approximate value of 30% for dU0 and dU(−)3. Additionally, the polymerization effectiveness order was found as follows: dU(−)5 > dU(−3) ~ dU0 > dU(+)3 > dU(+)5 > dU(+/−)5 > dU(+/−)3—in both discussed experiments. Interestingly, a comparative analysis of the polymerization assay between NE and NE with XRCC1 or NE/PCNA did not reveal evident differences. These results indicate that scaffold proteins are present in xrs5 NE at the correct/optimal level for other enzymes involved in BER.

Moreover, the polymerization differed for experiments with NE or pure Polβ (Figure 3B). These observations indicate that different polymerases are active, for example, for dU(−)5 and dU(+/−)5, the initiation of the long patch can be put forward, with a significant decrease in polymerization noted for dU(+)5 (see Figure 5A–C). 

Due to the above, the NE was supported in the additional Polβ amount/part (the same value was used for the pure enzyme experiment). As shown in Figure 5D, a reconstituted strand was observed for all the investigated *ds*-oligos; however, with different yields. The total yield of the repair was lower than in previous experiments (NE without Polβ) (Figure 4), which might have been caused by competition between polymerization and strand reconstitution. The repair efficiency was found in the following order: dU(−)3 > dU0 > dU(+)5 > dU(+/−)5 > dU(+/−)3 > dU(+)3 > dU(−)5. This observation indicates that pro-gapped *ds*-DNA (dU(+/−)5 and dU(+/−)3) were repaired more effectively than dU(−)5, for which the repair process via BER was barely observed, even with Polβ support. 

This phenomenon can be partially explained by the nature of the proteins involved in BER machinery. DNA repair via base excision is the sequence of the following events: recognition, removal, filling, and rejoining (Figure S2). Two of the initial steps are performed by proteins like UDG or hAPE1. These enzymes bind to the *ds*-DNA only from one side of the double helix. The situation becomes more complicated when strand elongation and reconstitution are considered. Most enzymes involved in these processes embrace the double helix in its three-dimensional model. Therefore, the *ds*-DNA is forced to move through proteins like a “bolt through the nut”. This leads to the situation where the surface interactions of the protein (in the internal part of an enzyme or at the place of DNA entry into enzyme structure) encounter a rigid structure (like cdPu), which renders DNA incapable of reaching the enzymes’ active site. Because of this, the whole repair process collapses. In these studies, the critical point of CDL repair was found for oligos where (5′*S*)cdA and SSB were separated by four nucleotides. As shown in Figure 4B, the presence of (5′*S*)cdA in the dU(−)5 position was not the stopping point for Polβ. The primer strand was extended by seven nucleotides, which was similar to the results found for the dU(−)3. The situation changed dramatically when the nuclear extract from the xrs5 cell line was investigated. The gapped *ds*-oligo dU(−)3 was reconstituted but dU(−)5 was not (Figure 5). However, in both cases, the polymerization assay had almost the same pattern as for the pure Polβ experiments. At this point, it should be mentioned, that during LP-BER, *ds*-DNA being under repair, penetrated PCNA (the clamp protein) and reached the polymerase δ/ε active site. Finally, the flipped-out oligo fragment containing (5′*S*)cdA in the descending strand became the substrate for FEN1 endonuclease. The structural changes forced by (5′*S*)cdA in the flipped-out strand could make it an unsuitable substrate for FEN1, leading to the inhibition of the whole process.

These results may lead to the assumption that in this case, an alternative mechanism was involved, as proposed in Figure 1B. This is supported by the work of Sweasy et al. [64] which has shown that Polβ can increase the rate of Okazaki fragment joining in Escherichia coli cells having a polymerase I defect. It should be pointed out that the polymerization efficiency of the discussed experiment (Figure 4D) was at the same level for all *ds*-oligos except dU(+)3. Additionally, these results are in good agreement with experiments concerning pure Polβ. These observations could become an interesting subject for future painstaking theoretical investigation (Molecular Dynamics).

## 3. Conclusions

The BER pathway was initiated by UDG, a specific glycosylase that recognizes DNA damage and incises it creating an AP-site. Next, the AP-site hydrolysis occurred, carried out, for example, by hAPE1 and leading to SSB formation. The gap formed this way in the *ds*-DNA was a suitable substrate for the polymerization reaction. Polβ is the most important polymerase in the base excision repair system due to its activity in both the SP- and LP-BER.

In this article, it has been shown that Polβ can initiate/support cdPu removal from the genome. It was found that the presence of (5′*S*)cdA in the descending oligomer exerts a negligible influence on the polymerization process (Figure 4A).

The Polβ assay performed for pro-gapped *ds*-oligos showed that dU(+/−)3 is not a suitable substrate for polymerization, due to the high flexibility of the *ss*-oligo fragment of five nucleotides located in the gap area. Extending the distance between two SSBs up to nine nucleotide units, in the case of dU(+/−)5 the pro-gapped oligo is stable enough to allow Polβ to elongate the primer strand by two dNMPs.

Since Polβ cooperated with the scaffold protein (XRCC1), the influence of the clustered DNA lesion structure (containing (5′*S*)cdA) on the polymerization process was verified. For the discussed *ds*-oligos, polymerization efficiency increased significantly. It indicates that XRCC1 cooperated with Polβ during its action upon the descending oligomer and accelerated movement of the template oligo through the active site of the enzyme.

The experiments involving Polβ and PCNA brought similar results to Polβ/XRCC1, despite showing slightly increased elongation of the primer strand.

The proposed hypothesis was validated during repair assays involving xrs5 nuclear extract. The results revealed that only for the control *ds*-oligo (dU0) and dU(−)3 strand reconstitution reached an acceptable level of ~30%, while for dU(−)5 an obscured ~5% was observed. Additionally, polymerase activity was higher in the case of dU(+)3 than dU(+)5, contrary to the results obtained from Polβ experiments. Moreover, the overall repair trends were unchanged after the addition of XRCC1 or PCNA from an external source.

In light of the above, the nuclear extract was supplemented with an additional amount of Polβ (artificial Polβ overexpression), and all oligonucleotides were reconstituted. The most effective repair was noted for dU(−)3, followed by dU(+/−)5.

These results indicated that (5′*S*)cdA was the substrate for BER proteins and may be effectively removed from the genome if it is a part of a single-stranded clustered DNA lesion.

## 4. Materials and Methods

For the experiments, the enzymes Polβ, PCNA, XRCC1, Lig1, Lig3 were obtained from Enzymax, LLC 870 Corporate Drive, Suite 201, Lexington, KY 40503, USA, while the enzymes UDG, hAPE1, T4 polynucleotide kinase were acquired from New England BioLabs, Ipswich, MA, USA, and [γ-^32^P]ATP was bought from Hartmann Analytic GmbH, Braunschweig, Germany. 

*Preparation of nuclear extracts*

The nuclear extracts (NE) from xrs5 cells were prepared according to the method previously described by Lomax et al. [26,39]. The NE was prepared from the xrs5 cell line (ATCC, CRL-2348, VA, USA), Ku80 deficient (this allows the avoidance of interference from Ku80 binding to linear DNA termini or SSBs) [61,62,63]. The xrs5 cells (CHO-K1 derived) were cultured in a MEM Alpha (Gibco) medium (15 mL) supplemented with 10% fetal bovine serum (BioWest). 

The xrs5 cells were harvested in the exponential phase with trypsin–EDTA and then centrifuged at 500× *g* for 5 min at 4 °C. The cell pellet was washed twice in 30 mL of phosphate-buffered saline. 

The cell pellet was treated using the NE-PER™ Nuclear and Cytoplasmic Extraction Reagents kit (no: #78835, ThermoFisher Scientific, Waltham, MA, USA) according to the manufacturer’s protocol. The concentration of NE was determined using a colorimetric Pierce™ 660 nm Protein Assay (ThermoFisher Scientific, Waltham, MA, USA) and was found in the range of 3.4–7.0 mg/mL. Aliquots of NE were stored at −80 °C for no longer than six months.

*Oligonucleotide synthesis, purification, and characterization*

The phosphoramidite derivatives of (5′S) 5′,8-cyclo-2′deoxyadenosine were synthesized as described previously by Romieu et al. [65]. All oligonucleotides (Figure 2) were synthesized and purified at the Bioorganic Chemistry Department, Polish Academy of Science, Lodz, Poland on a Geneworld (K and A Laborgeraete GbR, Schaafheim, Germany) synthesizer, using nucleotide phosphoramidites purchased from the ChemGenes Corporation. The crude oligonucleotides were purified by HPLC using Varian analytics with UV detection at wavelengths of λ = 260 nm, Phenomenex (Synergi 4 μm Fusion-RP 80Å, 250 × 4.6 mm) C-18 column. The oligonucleotides mass spectra were acquired in the negative-ion mode on a Waters Synapt G2-Si HDMS quadrupole time of flight hybrid mass spectrometer (Waters, Manchester, UK) at the Polish Academy of Science, Lodz, Poland. Data processing was performed with Waters MassLynx 4.1 software (deconvolution with the MaxEnt1 function). The calculated and found masses of the oligonucleotides were, respectively, as follows (calculated/found), 12,409.14/12,409.82 Matrix; 12,167.90/12,168.25 Cont.dU(0); 12,407.14/12,407.20 Cont.(cdA); 12,181.98/12,182.42 dU0; 12,165.90/12,166.30 dU(−)5; 12,165.90/12,166.46 dU(−)3; 12,165.90/12,166.25 dU(+)3; 12,180.90/12,181.54 dU(+)5; 12,166.89/12,167.30 dU(+/−)3; 12,166.89/12,167.20 dU(+/−)5. The adequate spectra scans are attached in the Supplementary Materials. The melting temperatures (Tm) of the *ds*-oligonucleotides were assigned on a Varian Cary 1.3E spectrophotometer, as described previously [31]. The found Tm values of *ds*-oligos were as follows [°C], 76.02 dU(0), 79.02 dU(−)5; 79.02 dU(−)3; 77.0 dU(+)3; 76.02 dU(+5); 77.22 dU(+/−)3; 77.02 dU(+/−)5.

*General procedures*

For all PAGE electrophoresis, a 15% or 20% denaturing polyacrylamide gel containing 8M urea in 1 × TBE [89 mM Tris-HCl, 89 mM boric acid, and 2 mM EDTA (pH 8.3)] was used.

For all biochemical reactions (except oligo radiolabeling), the following reaction buffer was used—70 mM Tris-HCl (pH 7.5), 10 mM MgCl_2_, 10 mM DTT, 4 mM ATP, 40 mM phosphocreatine, 1.6 μg ml^−1^ phosphocreatine kinase together with dATP, dCTP, dGTP, and dTTP (0.1 mM each).

To stop the reactions, 8 μL of loading buffer (LB) (98% formamide, 2 mM EDTA, 0.025% bromophenol blue, and 0.025% xylene cyanol) was added to all the samples, which were then subjected to electrophoresis. The results were visualized by autoradiography.

*Preparation of 5′-end-labelled oligonucleotides*

The oligonucleotides (0.2 μM) were 5′-end-labelled using 3.2 units of T4 polynucleotide kinase with 1.6 mCi (1.6 μL) [γ-^32^P]ATP in 16 μL of buffer (70 mM Tris-HCl (pH 7.6), 10 mM MgCl_2_, 100 mM KCl, and 1 mM β-2-mercaptoethanol) for 45 min at 37 °C. The protein denaturation was done by sample heating at 95 °C for 10 min. After incubation, the radiolabeled samples were precipitated with cold ethanol (200 μL) and kept at −80 °C for 30 min, centrifuged at 12,000 rpm for 30 min at 4 °C, and the remaining ethanol was removed under reduced pressure. The purity of the investigated oligonucleotides was examined on a 20% denaturing polyacrylamide gel (Figure S3).

*Double-stranded oligonucleotide with single-strand break formation* [31]

The 5′-end-labelled oligonucleotides were hybridized as follows: The required amount of radiolabeled oligonucleotide strand and complementary strand (0.14 μM and 0.21 μM, respectively) were dissolved in 80 μL of pure H_2_O and hybridized by heating at 90 °C for 10 min followed by slow cooling. 

*General procedure of single-strand break formation*

UDG and hAPE1 treatment of the *ds*-oligonucleotides presented in Figure 2 was as follows: 0.14 μM of each *ds*-oligonucleotide was dissolved in the required amount of reaction buffer (pH 7.9 at 37 °C) containing potassium acetate (50 mM), Tris-acetate (20 mM), magnesium acetate (10 mM), DTT (1 mM), and a mixture of digestion enzymes UDG and hAPE1 containing 4 units of each. The reactions were incubated at 37 °C for 60 min. After enzymatic digestion, each sample was precipitated with cold ethanol (250 μL) (vortexed, placed on dry ice for 30 min, and subsequently centrifuged at 13,000 rpm for 30 min at 4 °C). The ethanol was removed, and the residue was dried under reduced pressure at room temperature. The digestion efficiency and the purity of SSB-oligonucleotides were analyzed on the 20% denaturing (8M urea) PAGE. The purity of single and double-stranded oligonucleotides (Figure 2), as well as the purity of the formed SSB-DNA, are shown in Figure S3.

- *The influence of the presence of LMDS in ds-DNA on the strand-displacement activity of Polβ*

The polymerase strand-displacement activity was as follows: *ds*-oligonucleotides with SSB (3.63 pmol) were incubated with 0.26 pmol of Polβ in 8 μL of reaction buffer at 37 °C for 0, 1, 5, 10, and 15 min.

- *The effect of XRCC1 on Polβ activity on the strand-displacement activity of ds-DNA containing LMDS*

The polymerase strand-displacement activity in the presence of XRCC1 was as follows: The *ds*-oligonucleotides with SSB (3.63 pmol) were incubated with 0.26 pmol of Polβ and 0.21 pmol of XRCC1 in 8 μL of reaction buffer at 37 °C for 0, 1, 5, 10, and 15 min.

- *The effect of PNCA on Polβ activity on the strand-displacement activity of ds-DNA containing LMDS*

The polymerase strand-displacement activity in the presence of PCNA was as follows: the *ds*-oligonucleotides with SSB (3.63 pmol) were incubated with 0.26 pmol of Polβ and 0.32 pmol PCNA in 8 μL of reaction buffer at 37 °C for 0, 1, 5, 10, and 15 min.

- *The influence of the presence of LMDS in ds-DNA on strand-displacement and repair activity of xrs5 nuclear extract*

The *ds*-oligonucleotides (3.63 pmol) were incubated with 10 μg of xrs5 nuclear extracts in 8 μL of repair buffer at 37 °C for 0, 15, 30, 60, and 120 min.

- *The effect of PCNA (artificially supplemented) on xrs5 nuclear extract activity on the strand-displacement and repair activity of ds-DNA containing LMDS*

The *ds*-oligonucleotides (3.63 pmol) were incubated with 10 μg of xrs5 nuclear extracts together with 0.36 pmol of PCNA in 8 μL of repair buffer at 37 °C for 0, 15, 30, 60, and 120 min.

- *The effect of XRCC1 (artificially supplemented) on xrs5 nuclear extract activity on the strand-displacement and repair activity of ds-DNA containing LMDS*

The *ds*-oligonucleotides (3.63 pmol) were incubated with 10 μg of xrs5 nuclear extracts together with 0.21 pmol of XRCC1 in 8 μL of repair buffer mentioned above at 37 °C for 0, 15, 30, 60, and 120 min.

- *The effect of Polβ (artificially supplemented) on xrs5 nuclear extract activity on the strand-displacement and repair activity of ds-DNA containing LMDS*

The *ds*-oligonucleotides (3.63 pmol) were incubated with 10 μg of xrs5 nuclear extracts together with 0.26 pmol of Polβ in 8 μL of the repair buffer mentioned above at 37 °C for 0, 15, 30, 60, and 120 min. 

## Data Availability

All raw data have been shown in supplementary materials.

## Supplementary Materials

The following are available online at https://www.mdpi.com/article/10.3390/ijms22115934/s1, Figure S1. Types of DNA damage formed by the action of intercellular and environmental physical-chemical factors. Figure S2. Graphical representation of the base excision repair system divided into stages. Figure S3A. The radiograms presenting purity of A) single-stranded oligonucleotides, B) double-stranded oligonucleotides, C) double-stranded oligonucleotides after UDG and hAPE1 treatment. Figure S3B. The stability of Cont.cdA or matrix (double-stranded mode) in the given conditions. Figure S4. Graphical representations of the mutual positions of SSB and (5′S)cdA in a ds-DNA structure during Polβ action, and theoretical Polβ XRCC1position/interaction. Figure S5A. The activity of Ligase 3 in the presence of XRCC1 and Polβ on cluster lesioned ds-DNA strand ligation. Figure S5B. The activity of Ligase 1 in the presence of XRCC1 and Polβ on cluster lesioned ds-DNA strand ligation. Figure S5C. The activity of Ligase 1 in the presence of PCNA and Polβ on cluster lesioned ds-DNA strand ligation. Figure S6A. Three independent experiments presenting the oligonucleotide’s stability radiograms. Figure S6B. Three independent experiments presenting the influence of clustered lesion on Polβ strand displacement. Figure S6C. Three independent experiments presenting the influence of XRCC1 on strand displacement by Polβ. Figure S6D. Three independent experiments presenting the influence of PCNA on the strand displacement by Polβ. Figure S7A. Three independent experiments presenting SSB rejoining, formed from ds-oligonucleotide presented in Figure 2, after UDG and hAPE1 digestion, by nuclear extract of xrs5 cells. Figure S7B. Three independent experiments presenting SSB rejoining, formed from ds-oligonucleotide presented in Figure 2, after UDG and hAPE1 digestion, by nuclear extract of xrs5 cells in the PCNA presence from an external source. Figure S7C. Three independent experiments presenting SSB rejoining, formed from ds-oligonucleotide presented in Figure 2, after UDG and hAPE1 digestion, by nuclear extract of xrs5 cells in the XRCC1 presence from an external source. Figure S7D. Three independent experiments presenting SSB rejoining, formed from ds-oligonucleotide presented in Figure 2, after UDG and hAPE1 digestion, by nuclear extract of xrs5 cells in the Polβ presence from an external source. Table S1. The raw data presented by graphs in Figure 4. Average and corresponding standard deviations values. In the final section of the Supplementary Materials, the spectra of oligonucleotide mass spectroscopy analysis is presented.

## Abbreviations

(5′*S*)cdA—(5′*S*) 5′,8-cyclo-2′-deoxyadenosine, cdPu—5′,8-cyclo-2′-deoxyPurines, SSB—single-strand break, DSB—double-strand break, ^oxo^dG—7,8-dihydro-8-oxo-2′-deoxyGuanosine, dU—2′-deoxyuridine, LMDSs—local multiple damage sites, AP-site—apurinic/apyrimidinic site, BER—base excision repair, SP-BER—short patch BER, LP-BER—long patch BER, NER—nucleotide excision repair mechanism, Polβ—polymerase beta, Polε—polymerase epsilon, Polδ—polymerase delta, PCNA—proliferating cell nuclear antigen, XRCC1—x-ray repair cross-complementing protein one, Lig1—ligase one, Lig3—ligase three, UDG—uracil-DNA glycosylase, hAPE1—human AP site nuclease one, FEN1—flap endonuclease one, PAGE—PolyAcrylamide Gel Electrophoresis, dNTP—2′-deoxynucleosides triphosphate, NE—nuclear extract, xrs5—x-ray sensitive Chinese hamster ovary mutant cell line.
