# Supplementary material for: (5′S) 5′,8-cyclo-2′-deoxyadenosine Cannot Stop BER. Clustered DNA Lesion Studies
Source: Int J Mol Sci. 2021 May 31;22(11):5934. doi: 10.3390/ijms22115934 (PMC8199134; doi:10.3390/ijms22115934)

Supplementary Materials

(5′*S*) 5′,8-cyclo-2′-deoxyAdenosine can't stop BER. Clustered DNA lesion studies.

Boleslaw T. Karwowski*

DNA Damage Laboratory of Food Science Department, Faculty of Pharmacy,

Medical University of Lodz, ul. Muszynskiego 1, 90-151 Lodz

Email: Boleslaw.Karwowski@umed.lodz.pl

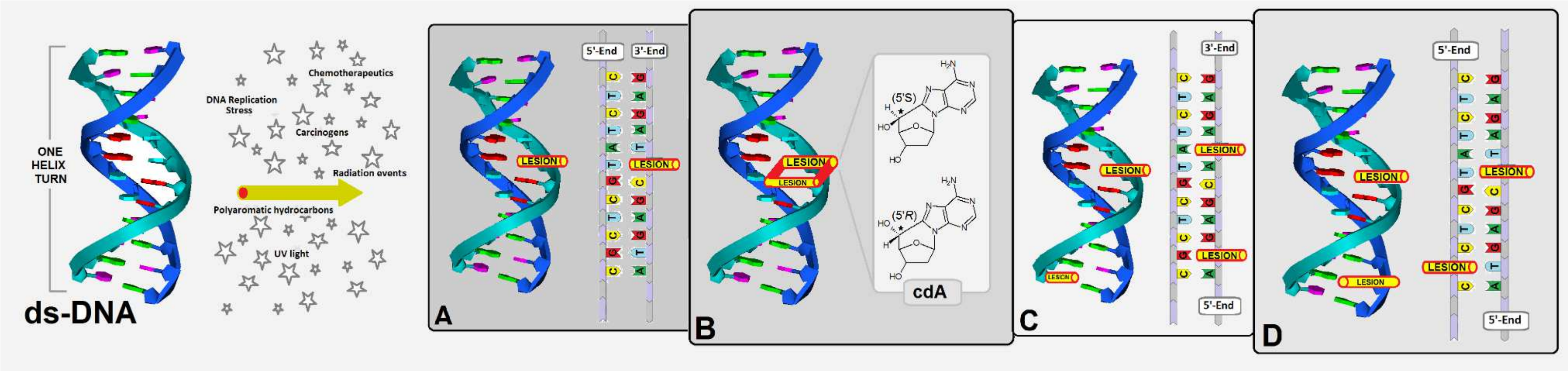

Figure S1. Types of DNA damage formed by the action of intercellular and environmental physical-chemical factors: **A**) isolated lesion - one per one or two helix turn, **B**) tandem lesions - is a particular type of clustered DNA damage consisting two contiguously damaged nucleosides or consisted of two adjacent modifications, e.g., sugar and base moieties, resulting from a single free radical initiating event, **C**) single-stranded DNA clustered lesion - two or more DNA damage located in the same oligonucleotide strand of ds-DNA per one or two double helix turn, **D**) double-stranded DNA clustered lesion - two or more DNA damage present in both ds-DNA complementary strands: matrix and complementary, per one or two double helix tur

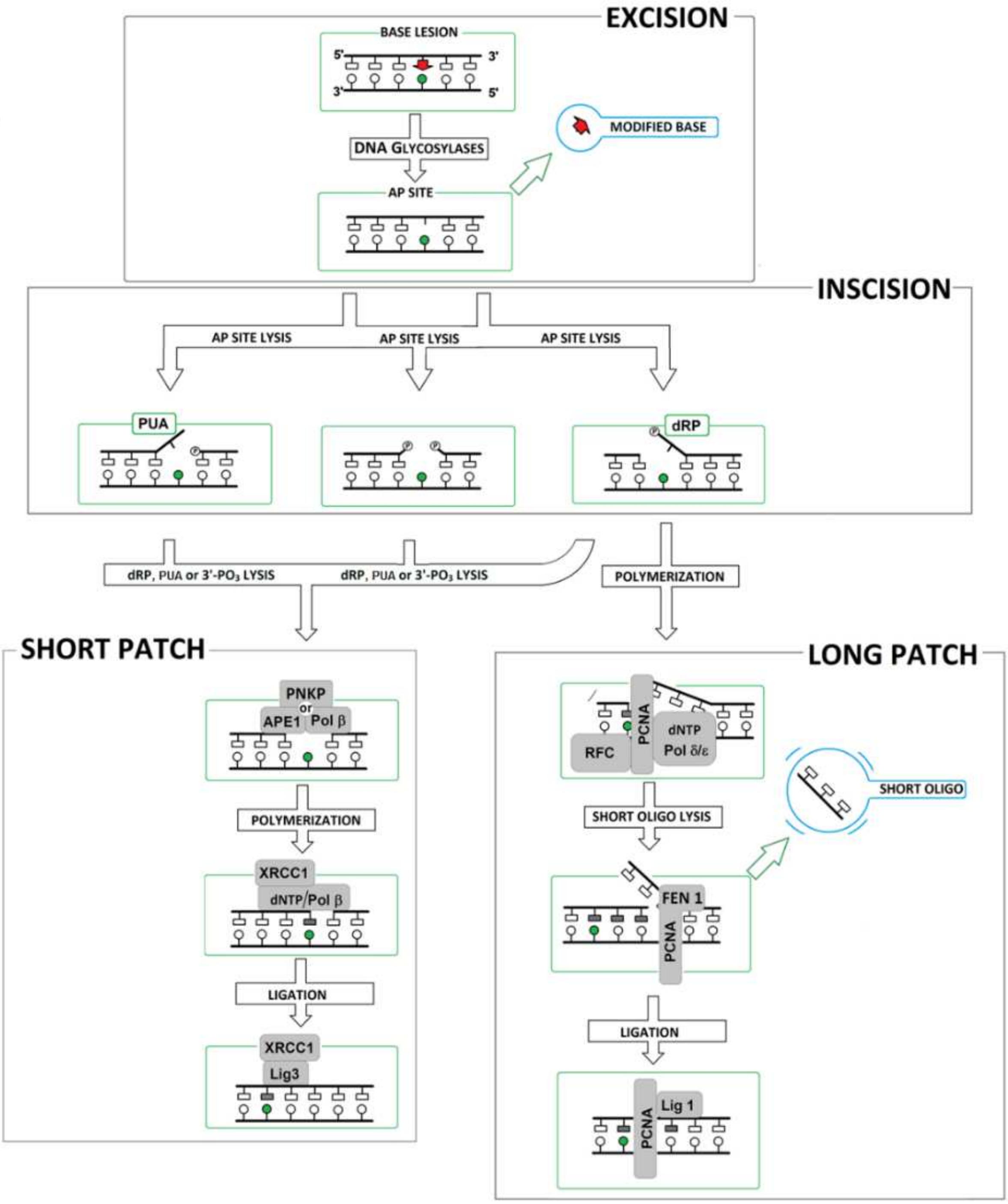

Figure S2. Graphical representation of the **B**ase **E**xcision **R**epair system divided to stages.

Apurinic/Apyrimidinic site (**AP-site**), 3′-Phospho-α,β-Unsaturated Aldehyde (**PUA**), phosphate group (**P**), 5′-deoxyRibose Phosphate (**dRP**), PolyNucleotide Phosphatase/Kinase (**PNKP**), AP endonuclease one (**APE1**), polymerase beta (**Polβ**), deoxynucleotide triphosphates **(dNTP**), X-ray repair cross-complementing protein 1 (**XRCC1**), ligase one (**Lig1**), ligase three (**Lig3**), replication factor-C (**RFC**), polymerase delta (**Polδ),** polymerase epsilon (**Polε**), flap endonuclease one (**FEN1**), Proliferating Cell Nuclear Antigen (**PCNA**).

Figure S3A.

Radiograms of:

A) Purity of single-stranded oligonucleotides as follows: **1**–matrix, **2**–dU0, **3**–dU(-)5, **4**–dU(-)3, **5**–dU(+/-)3, **6**–dU(+/-)5, **7**–dU(+)3, **8**–dU(+)5; 20% polyacrylamide with 8M urea gel electrophoresis.

B) Purity of double-stranded oligonucleotides as follows: **1**–dU0, **2**–dU(-)5, **3**–dU(-)3, **4**–dU(+/-)3, **5**–dU(+/-)5, **6**–dU(+)3, **7**–dU(+)5; 20% polyacrylamide gel (native) electrophoresis.

C) Purity of double-stranded oligonucleotides after UDG and hAPE1 digestion with subsequent precipitation: **1**–matrix, **2**–dU(+)5, **3**–dU(+)3, **4**–dU(+/-)5, **5**–dU(+/-)3, **6**–dU(-)3, **7**–dU(-)5, **8**–dU0 20% polyacrylamide with 8M urea gel electrophoresis.

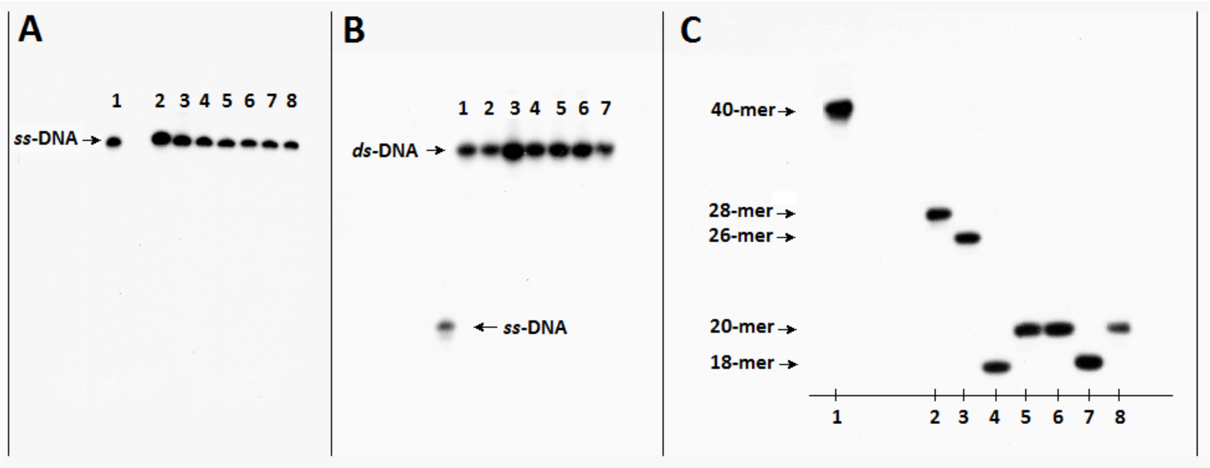

Figure S3B.

The stability of Cont. (5′*S*)cdA or Matrix (double-stranded mode) in the following conditions:

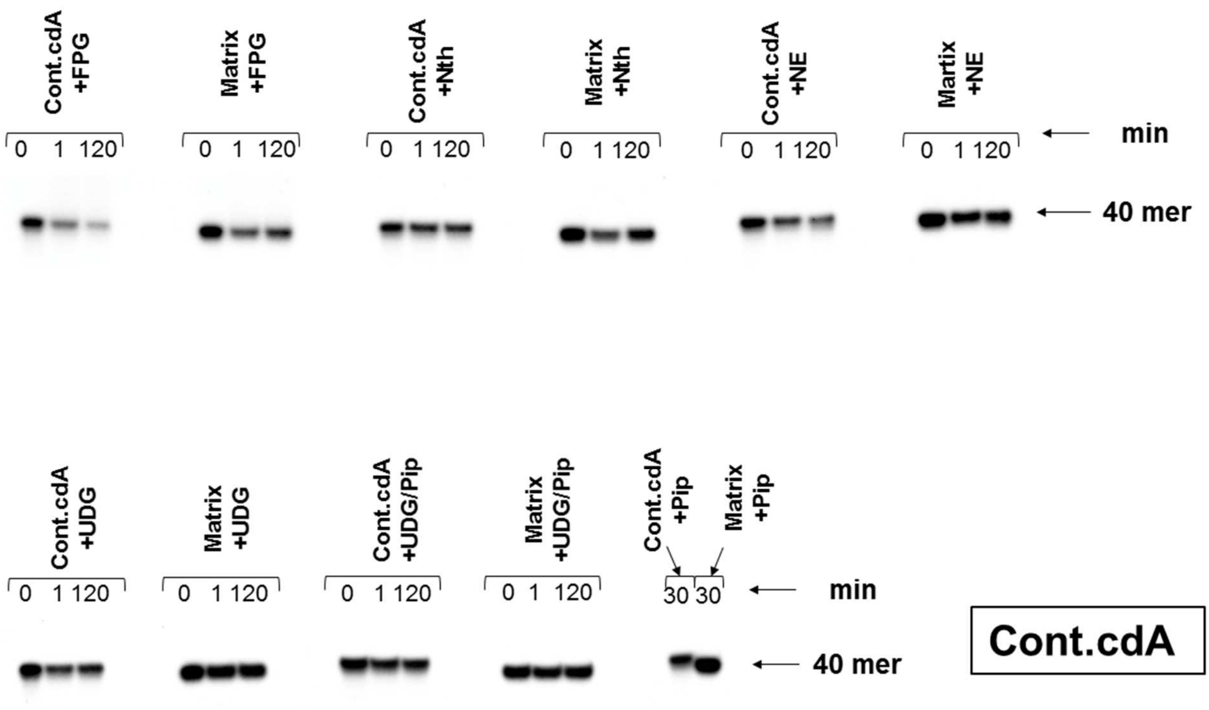

1.1. Nuclear Extracts

The shortcut cdA corresponding to (5′*S*) 5′,8-cyclo-2′-deoxyadenosine.

10 μg of xrs5 **NE** was incubated with Cont.cdA or Matrix in 8 μL of repair buffer at 37° C for the following times (in minutes): 0, 1 and 120. After the required time, the reactions were stopped with 8 μL of loading buffer (denaturing) and the samples were examined on a 15% denaturing polyacrylamide gel.

1.2. FGP, Nth, and UDG

5U of **FPG** was incubated with Cont.cdA or Matrix in 5 μL of reaction buffer (10 mM Bis-Tris Propane-HCl (pH 7.0), 10 mM MgCl2, 1 mM DTT, and 100 μg/ml BSA) at 37 ° C for 0, 1 and 120 min.

5U of **Nth** was incubated with Cont.cdA or Matrix in 5 μL of reaction buffer (20 mM Tris-HCl (pH 8.0), 1 mM EDTA and 1mM DTT) at 37°C for 0, 1 and 120 minutes.

5U of **UDG** was incubated with Cont.cdA or Matrix in 5 μL of reaction buffer (20 mM Tris-HCl (pH 8.0), 1 mM EDTA and 1mM DTT) at 37°C for 0, 1 and 120 minutes.

After the required time, the reactions were stopped with 5 μL of loading buffer and the samples were examined on a 15% denaturing polyacrylamide gel.

1.3. Hot 1M piperidine

Cont.cdA or Matrix was incubated with 100 μL of **1M piperidine** at 80°C for 30 min. After the required time, the samples were precipitated with 250 μL of cold ethanol and 2 μL of glycogen (placed on dry ice, 30 minutes), centrifuged (13,000 rpm, 4°C, 30 minutes) and dried under reduced pressure at room temperature. The residues were resuspended in 5 μL of loading buffer and the samples were examined on a 15% denaturing polyacrylamide gel.

1.4. UDG and 1M piperidine

5U of **UDG** was incubated with Cont.cdA or Matrix in 5 μL of reaction buffer (20 mM Tris-HCl (pH 8.0), 1 mM EDTA and 1mM DTT) at 37°C for 0, 1 and 120 minutes. After the required time, the reactions were stopped by placing samples on ice (4°C) and subsequently incubated with 100 μL of **1M piperidine** at 80°C for 30 minutes. After this time, the samples were precipitated with 250 μL of cold ethanol and 2 μL of glycogen (placed on dry ice, 30 min), centrifuged (13,000 rpm, 4°C, 30 min) and dried under reduced pressure at room temperature. The residues were resuspended in 5 μL of loading buffer and the samples were examined on a 15% denaturing polyacrylamide gel.

Figure S4. Graphical representations of the SSB and (5′*S*)cdA mutual positions in a *ds*-DNA structure during Polβ action, and theoretical Polβ XRCC1 position/interaction, based on work of London and Pelletire **[**Cuneo, M.J.; London, R.E. (2010). The oxidation state of the XRCC1 N-terminal domain regulates DNA polymerase β binding affinity. PNAS., **107**, 6805–6810; Sawaya M.R., Prasad R., Wilson S.H., Kraut J., Pelletier H. (1997) Crystal Structures of human DNA polymerase β complexed with gapped and Nicked DNA: evidence for an induced fit mechanism. Biochemistry, **36**, 11205-11215].

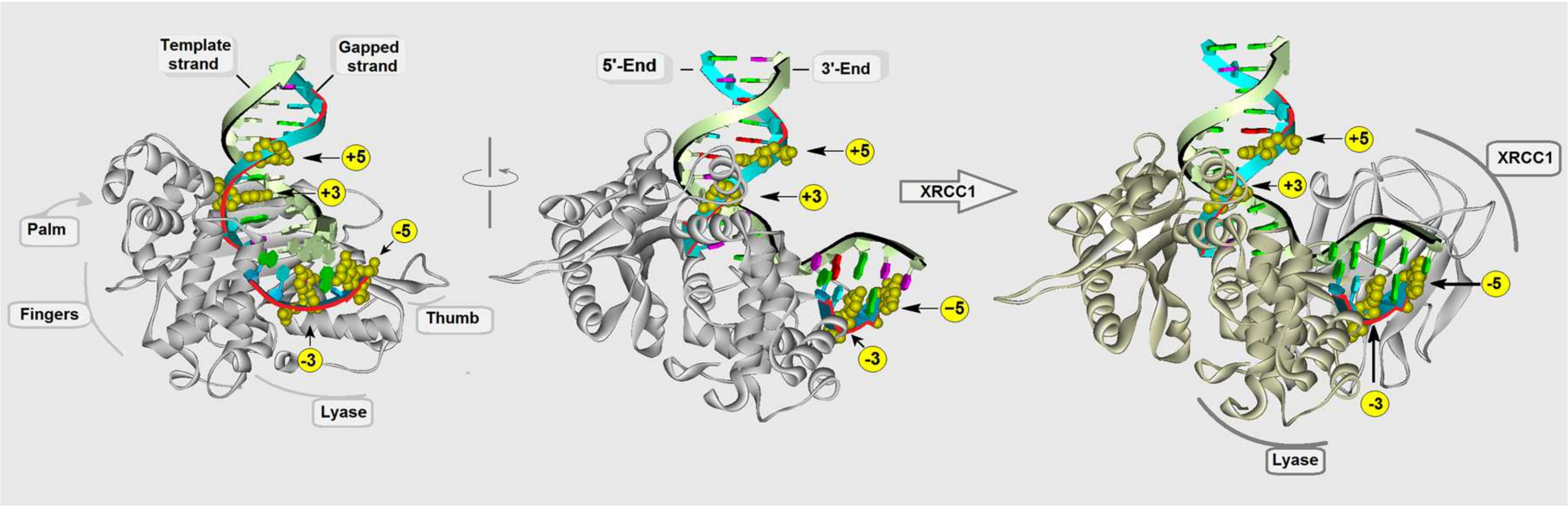

Figure S5A. Activity of Lig3 in the presence of XRCC1 and Polβ on cluster lesioned *ds*-DNA strand ligation. For each lane/sample (8 μl): 3.63 pmol of suitable *ds*-oligonucleotide with SSB (**Table 1**), 0.26 pmol of Polβ, 0.21 pmol of XRCC1 and 0.51 pmol of Lig3 were used in the reaction buffer (70 mM Tris-HCl (pH 7.5), 10 mM $MgCl_2$, 10 mM DTT, 4 mM ATP, 40 mM phosphocreatine, 1.6 μg ml^{-1} phosphocreatine kinase together with dATP, dCTP, dGTP, and dTTP (0.1 mM each).

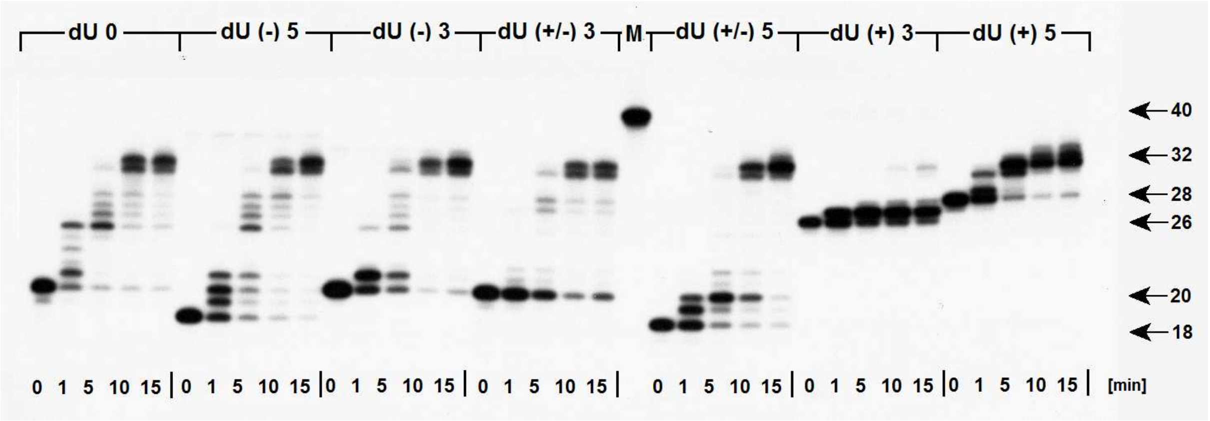

Figure S5B. Activity of Lig1 in the presence of XRCC1 and Polβ on cluster lesioned *ds*-DNA strand ligation. For each lane/sample (8 μl): 3.63 pmol of suitable *ds*-oligonucleotide with SSB (**Table 1**), 0.26 pmol of Polβ, 0.21pmol of XRCC1 and 0.56 pmol of Lig1 were used in the reaction buffer (70 mM Tris-HCl (pH 7.5), 10 mM $MgCl_2$, 10 mM DTT, 4 mM ATP, 40 mM phosphocreatine, 1.6 μg ml^{-1} phosphocreatine kinase together with dATP, dCTP, dGTP, and dTTP (0.1 mM each).

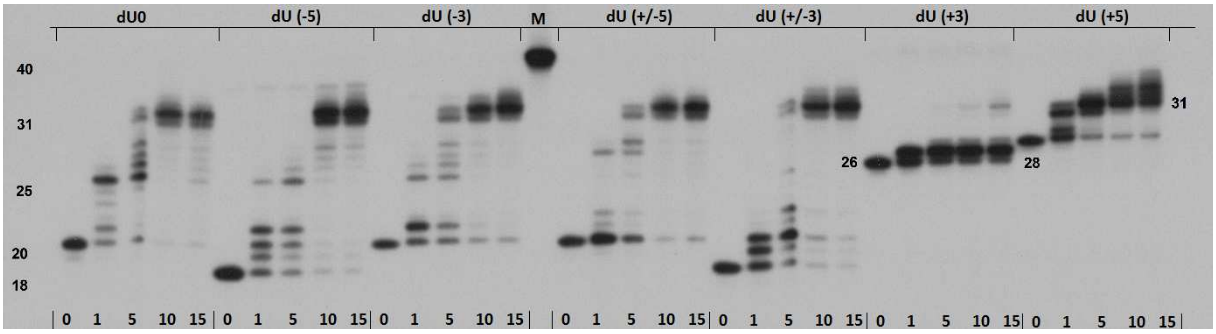

Figure S5C. Activity of Lig1 in the presence of PCNA and Polβ on cluster lesioned *ds*-DNA strand ligation. For each lane/sample (8 µl): 3.63 pmol of suitable *ds*-oligonucleotide with SSB (Table 1), 0.26 pmol of Polβ, 0.32 pmol of PCNA and 0.56 pmol of Lig1 were used in the reaction buffer (70 mM Tris-HCl (pH 7.5), 10 mM $MgCl_2$, 10 mM DTT, 4 mM ATP, 40 mM phosphocreatine, 1.6 µg ml^{-1} phosphocreatine kinase together with dATP, dCTP, dGTP, and dTTP (0.1 mM each).

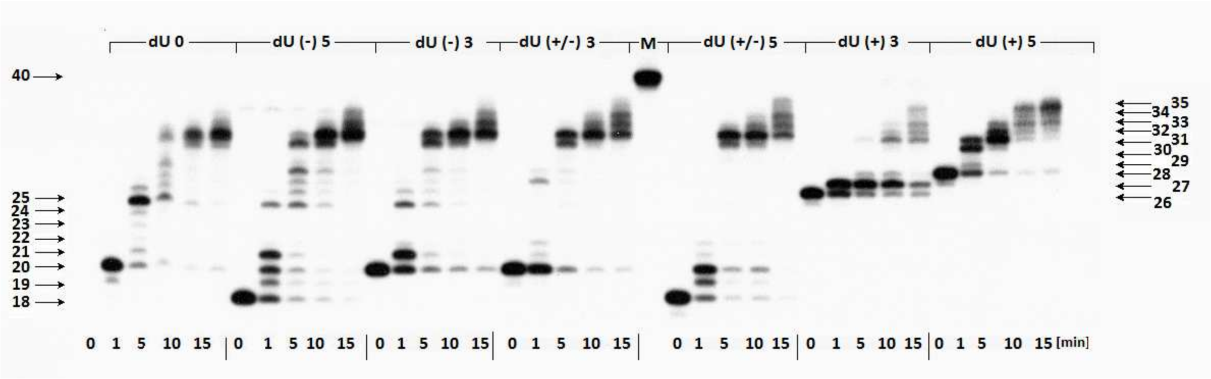

Figure S6A. Three independent experiments of oligonucleotide stability radiograms presented in **Figure 2A** and fully described (ibid.).

Experiment 1

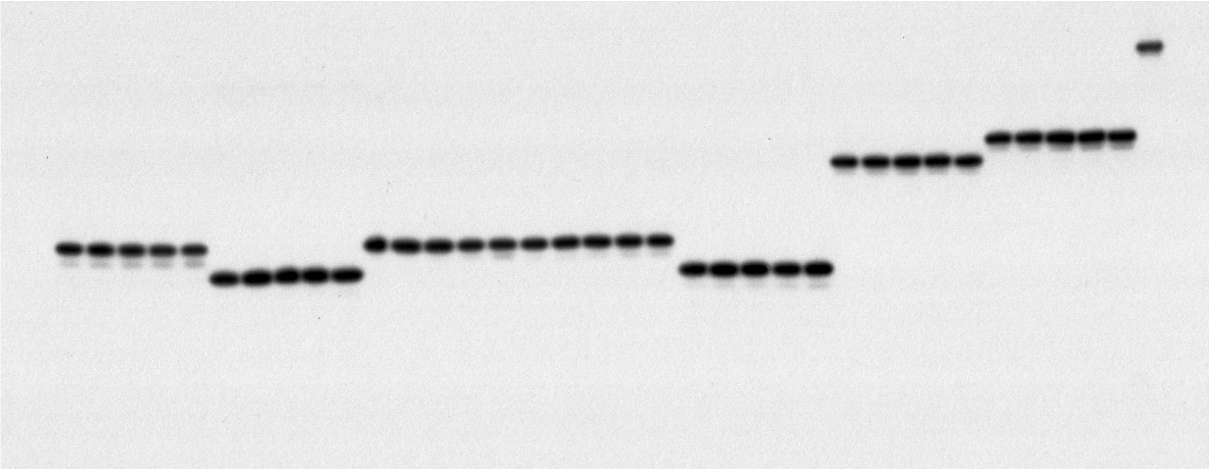

Experiment 2

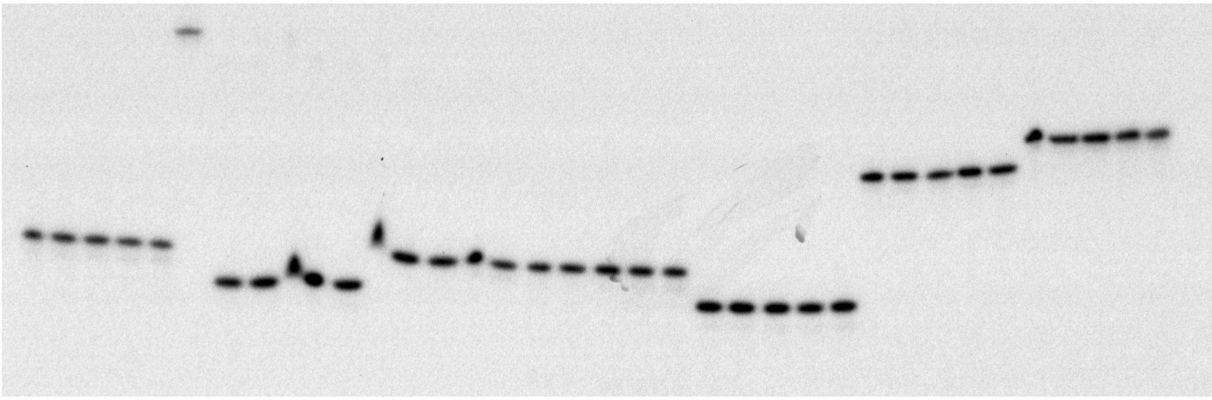

Experiment 3

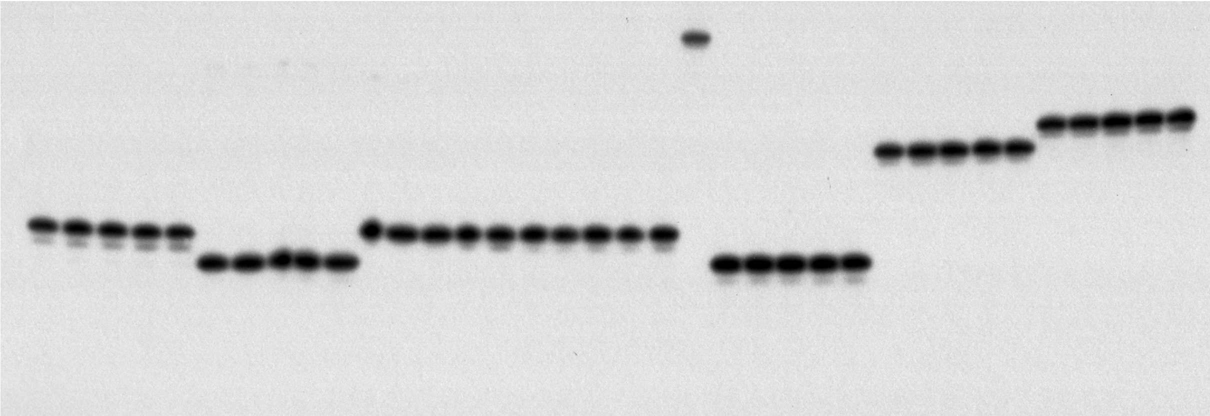

Figure S6B. Three independent experiments on the influence of a clustered lesion on Polβ strand-displacement presented in **Figure 2B** and fully described (ibid.).

Experiment 1

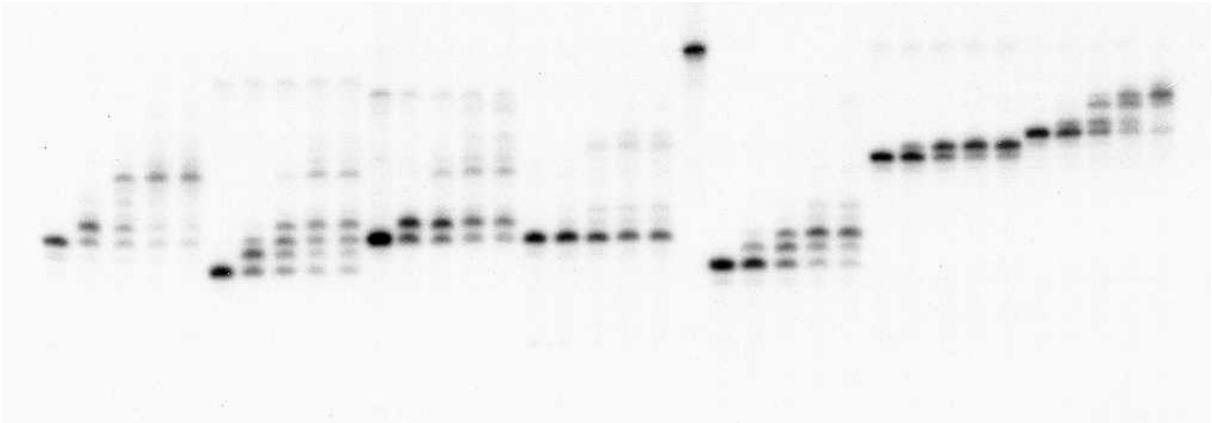

Experiment 2

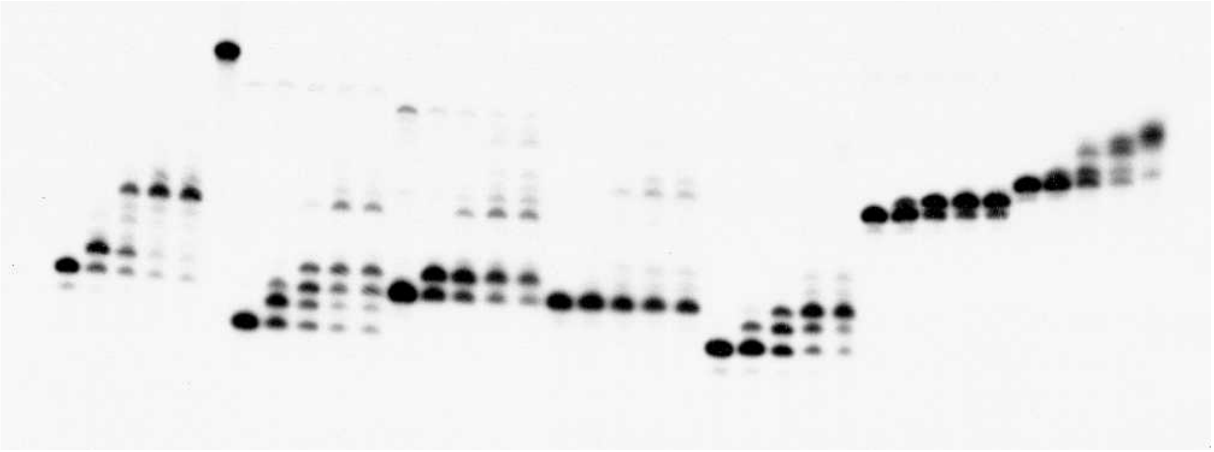

Experiment 3

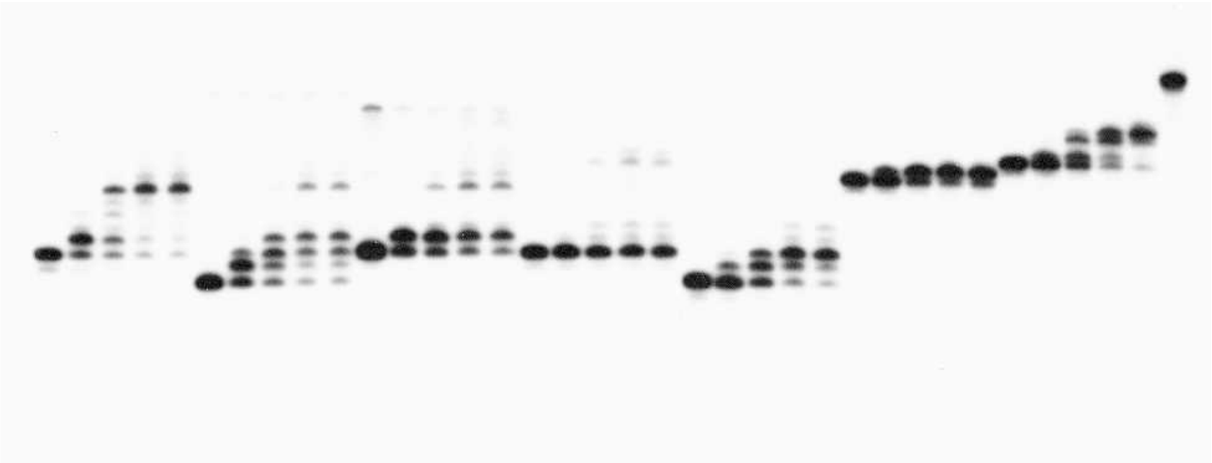

Figure S6C. Three independent experiments on the influence of XRCC1 on strand displacement by Polβ presented in **Figure 2C** and fully described (ibid.).

Experiment 1

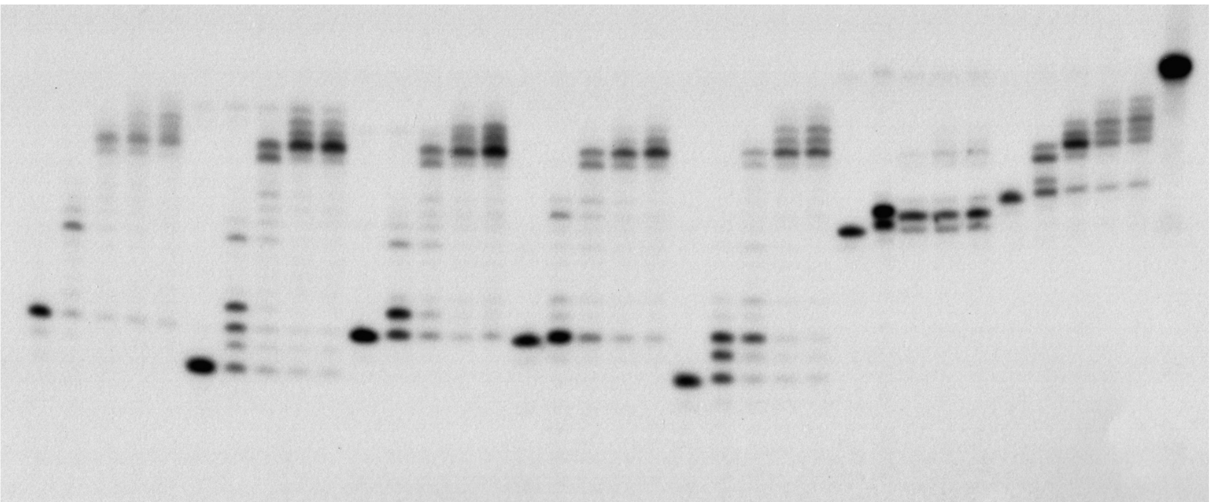

Experiment 2

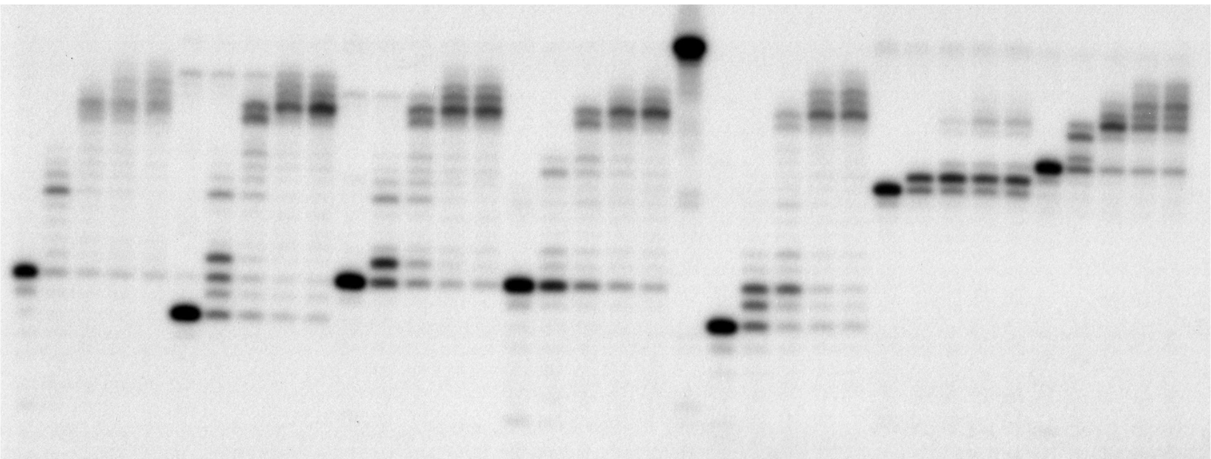

Experiment 3

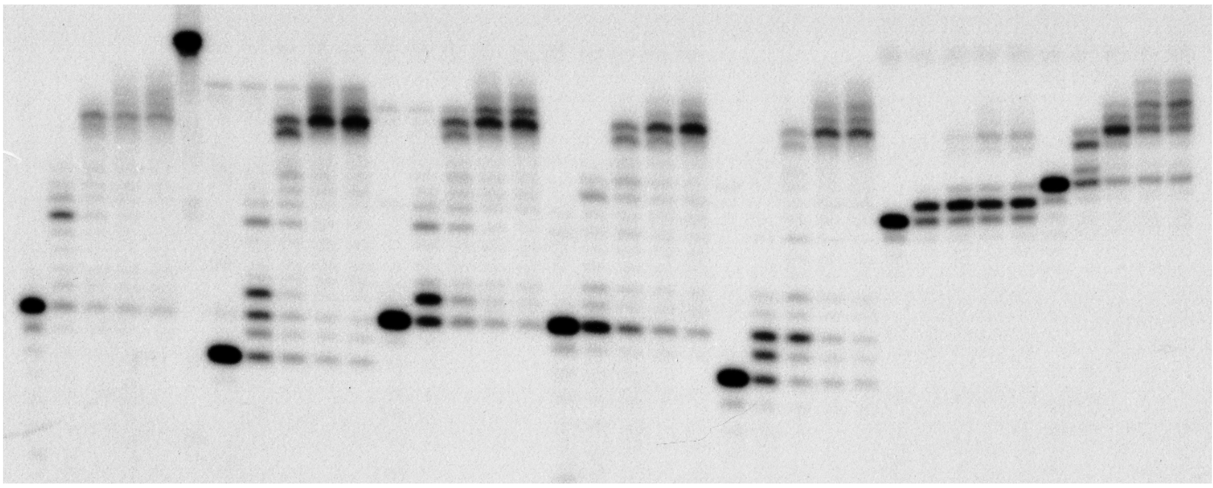

Figure S6D. Three independent experiments on the influence of PCNA on strand displacement by Polβ presented in **Figure 2D** and fully described (ibid.).

Experiment 1

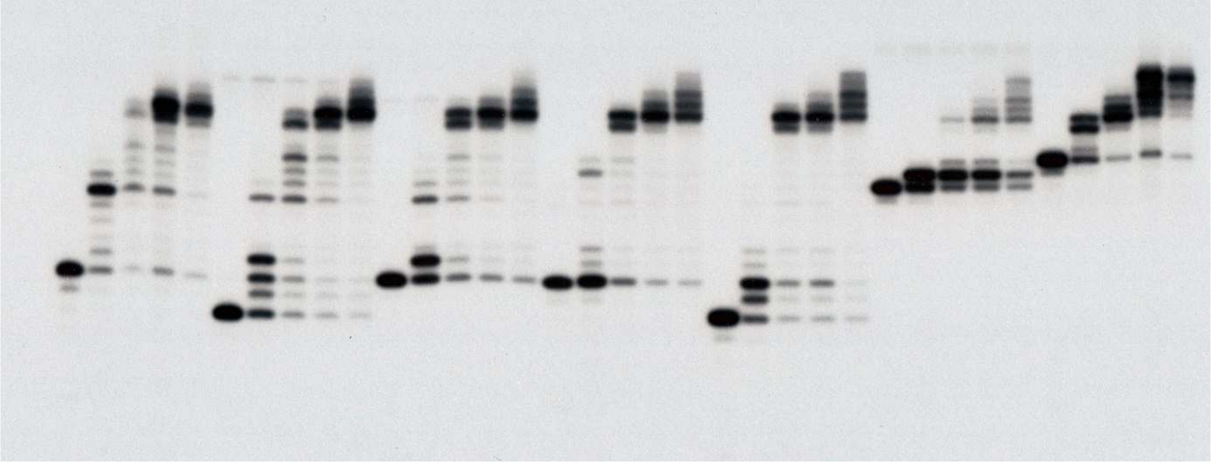

Experiment 2

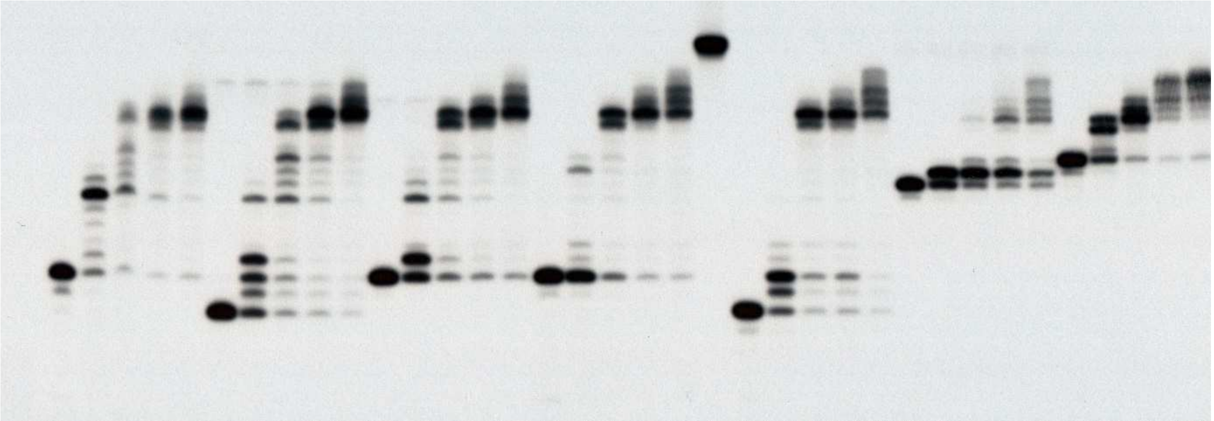

Experiment 3

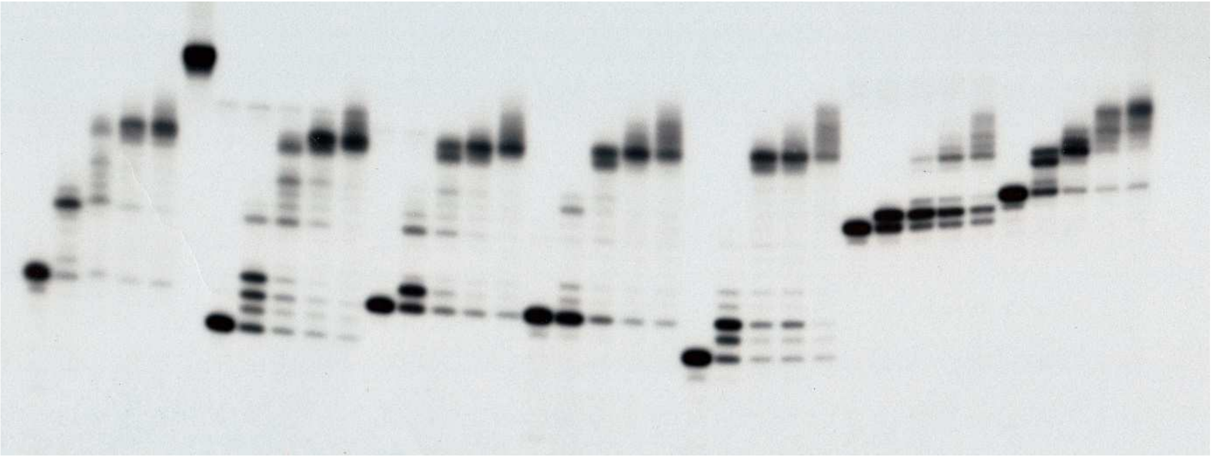

Figure S7A. Three independent experiments of SSB rejoining, formed from *ds*-oligonucleotide presented in **Table 1**, after UDG and HAPE1 digestion, by nuclear extract of xrs5 cells.

Experiment 1

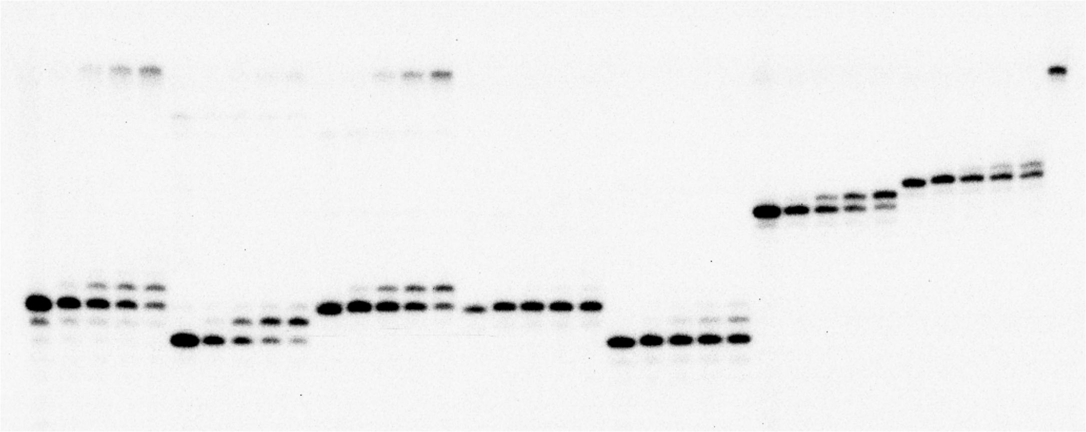

Experiment 2

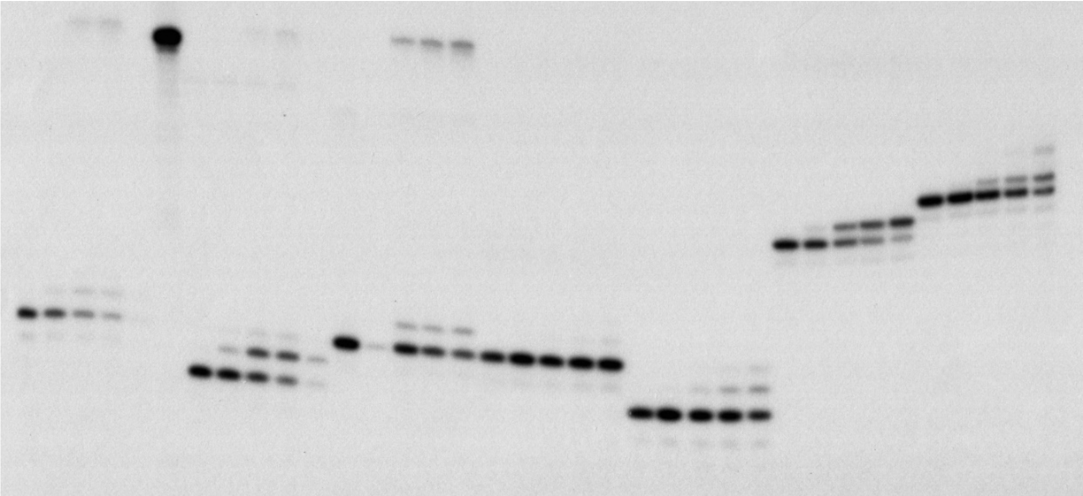

Experiment 3

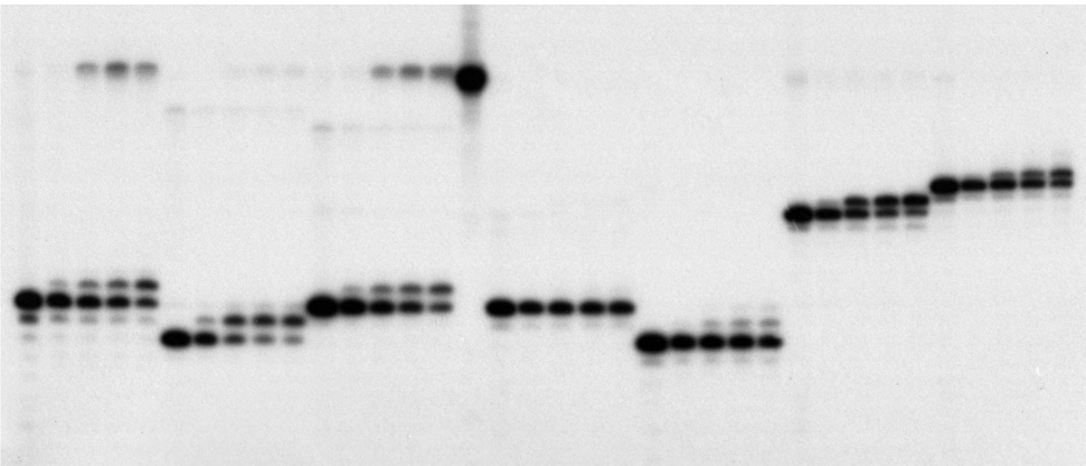

Figure S7B. Three independent experiments of SSB rejoining, formed from *ds*-oligonucleotide presented in **Table 1**, after UDG and hAPE1 digestion, by nuclear extract of xrs5 cells in the PCNA presence of an external source.

Experiment 1

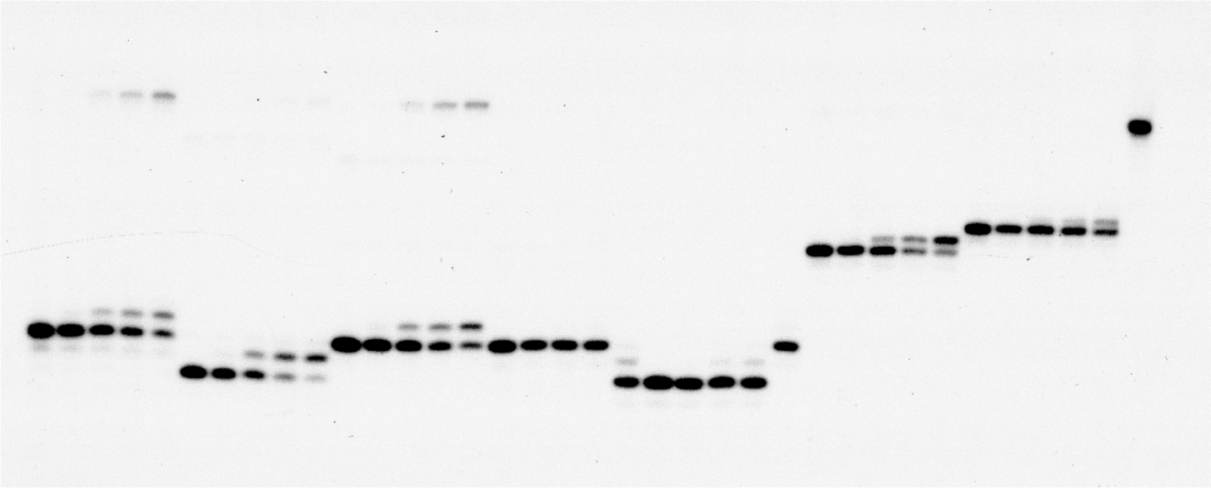

Experiment 2

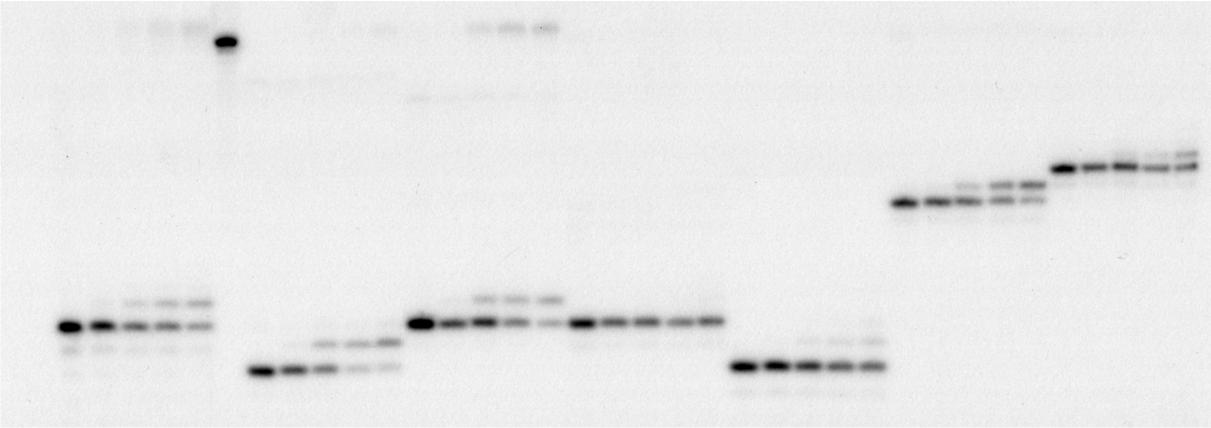

Experiment 3

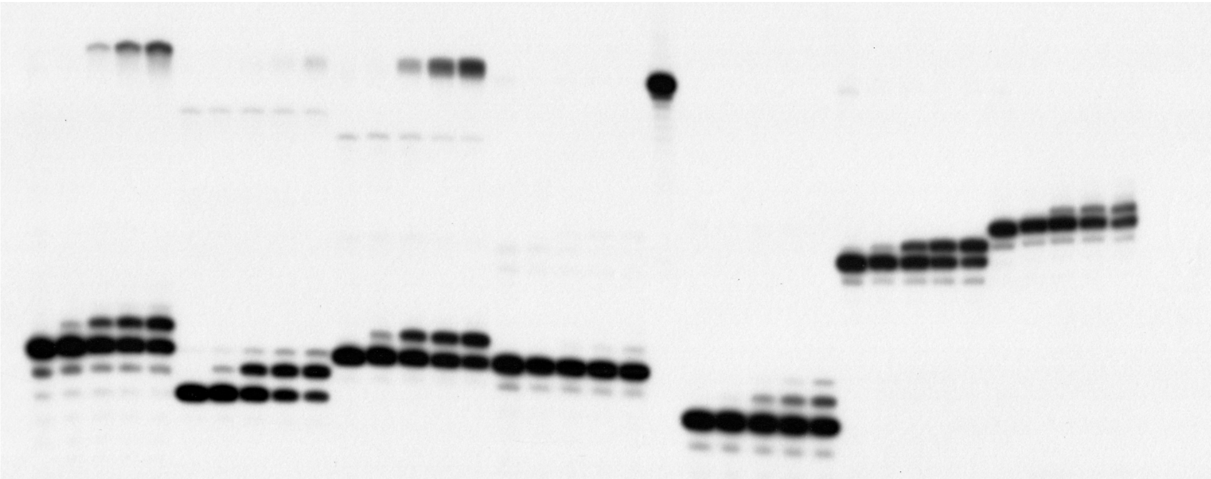

Figure S7C. Three independent experiments of SSB rejoining, formed from *ds*-oligonucleotide presented in **Table 1**, after UDG and hAPE1 digestion, by nuclear extract of xrs5 cells in the XRCC1 presence of an external source.

Experiment 1

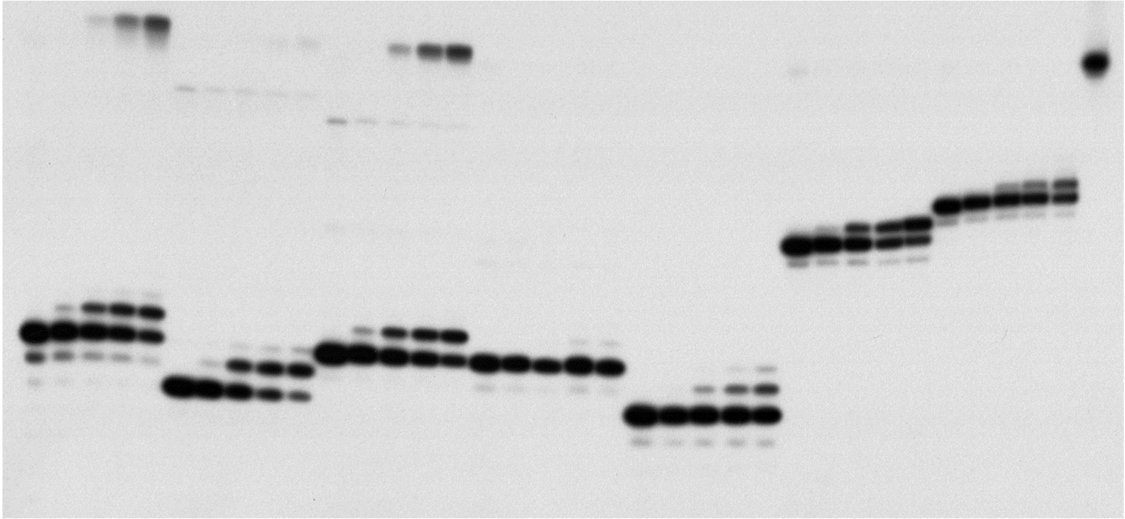

Experiment 2

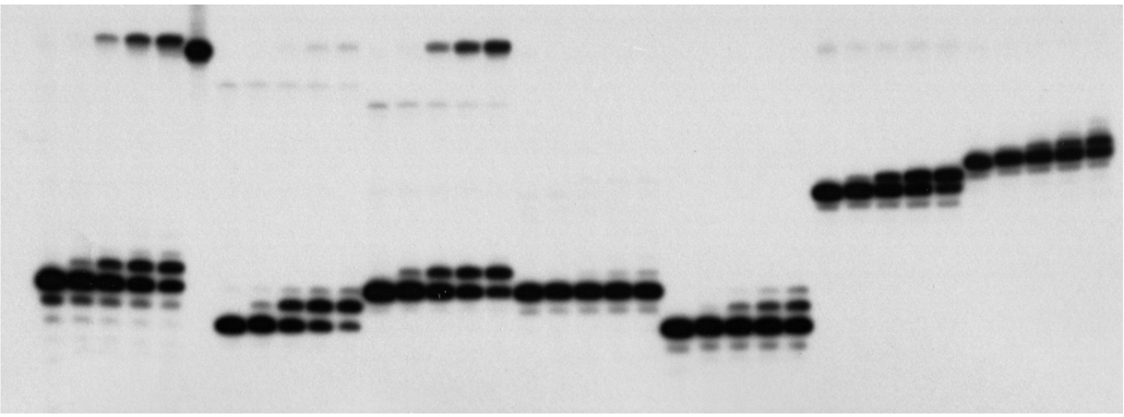

Experiment 3

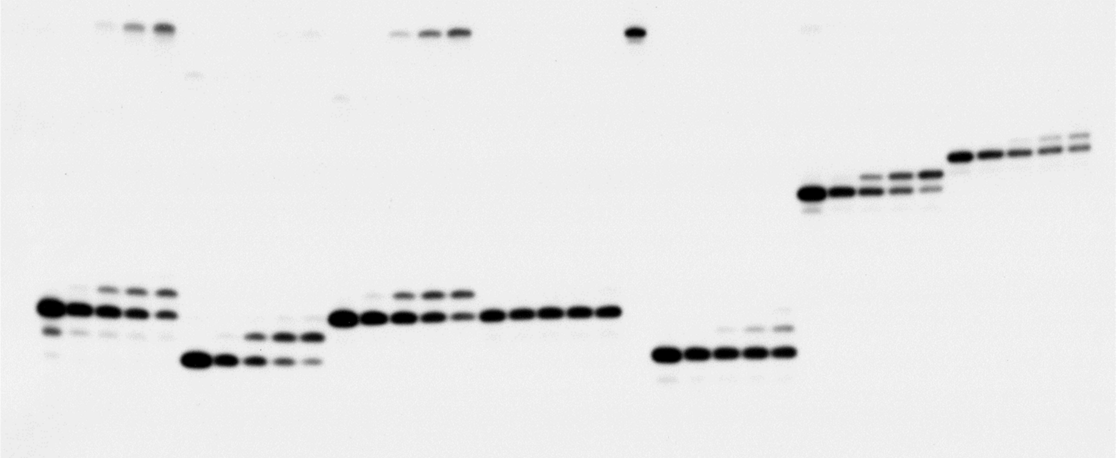

Figure S7D. Three independent experiments of SSB rejoining, formed from *ds*-oligonucleotide presented in Table 1, after UDG and hAPE1 digestion, by nuclear extract of xrs5 cells in the Polβ presence of an external source.

Experiment 1

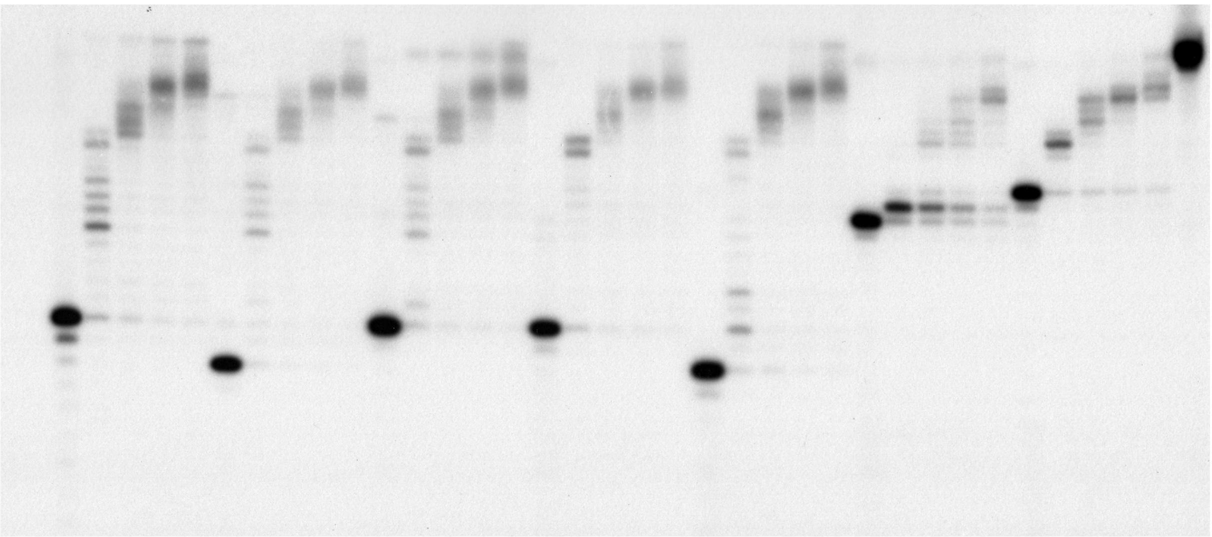

Experiment 2

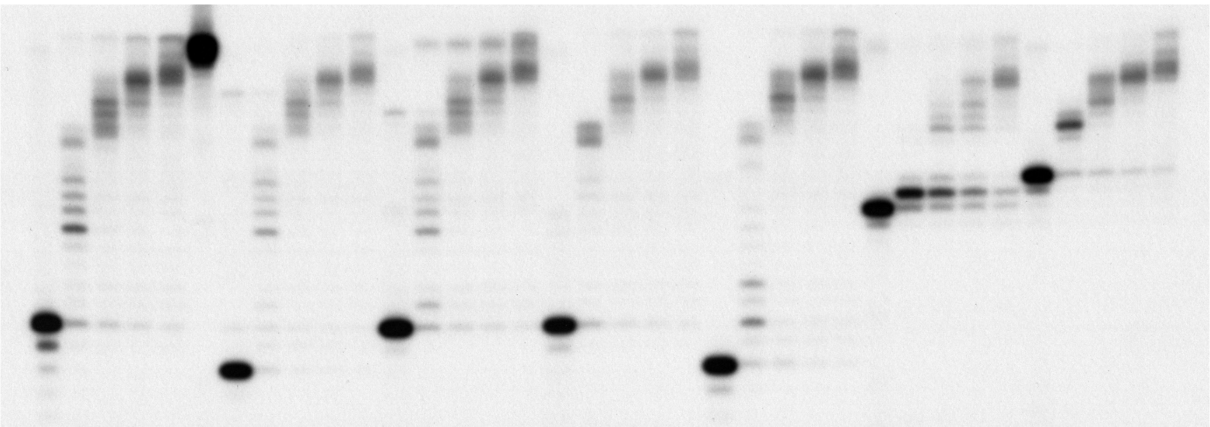

Experiment 3

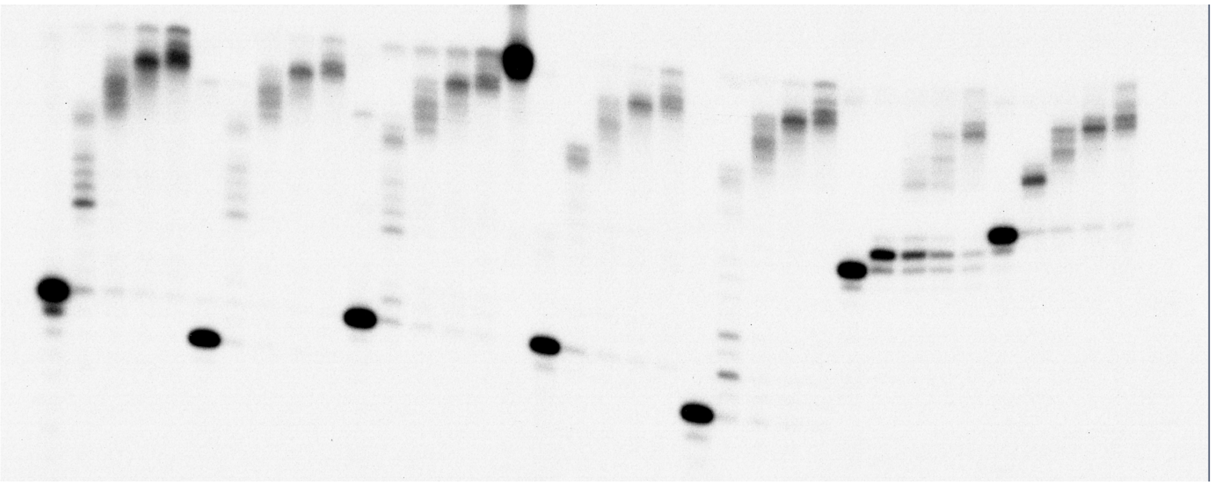

Table S1. The raw data presented by graphs in **Fig. 3**. Average and corresponding standard deviations values.

Time scale of strand elongation contained in primer or descending strand clustered lesion hybridized to non-damaged template of *ds*-DNA, after UDG and hAPE1 digestion, by:

A) exclusively Polβ

Time [min]	dU"0"	dU(-5)	dU(-3)	dU(+/-)3	dU(+/-)5	dU(+)3	dU(+)5
**Experiment 1**	**Percentage of Digestion [%]**						
0	0	0	0	0	0	0	0
1	64.97	68.51	50.48	0	41.80	39.17	68.49
5	82.96	80.09	61.83	15.66	67.79	53.043	68.52
10	86.81	85.35	73.74	16.19	81.80	59.00	79.68
15	87.55	86.65	74.63	17.60	86.89	54.32	90.20
**Experiment 2**	**Percentage of Digestion [%]**						
0	0	0	0	0	0	0	0
1	72.867	65.69	52.53	0	44.30	35.59	58.59
5	82.63	79.27	63.57	18.85	71.16	52.42	70.24
10	85.43	83.44	77.22	20.50	82.55	59.58	78.75
15	84.68	84.72	78.15	23.14	84.96	60.86	82.88
**Experiment 3**	**Percentage of Digestion [%]**						
0	0.00	0.00	0.00	0.00	0.00	0.00	0.00
1	66.97	63.67	51.92	0.00	45.31	54.28	65.11
5	81.50	78.70	60.10	25.14	68.69	54.00	60.74
10	86.04	85.71	71.40	36.60	81.90	57.39	73.96
15	86.36	87.62	74.21	33.92	86.58	53.57	79.73
**Time [min]**	**Average Percentage of Digestion [%]**						
0	0.00	0.00	0.00	0.00	0.00	0.00	0.00
1	68.27	65.96	51.64	0.00	43.80	43.01	64.06
5	82.37	79.35	61.83	19.88	69.21	53.15	66.50
10	86.09	84.83	74.12	24.43	82.08	58.65	77.46
15	86.20	86.33	75.67	24.89	86.15	56.25	84.27
**Standard Deviation**							
0	0.00	0.00	0.00	0.00	0.00	0.00	0.00
1	4.11	2.43	1.05	0.00	1.81	9.92	5.03
5	0.77	0.70	1.73	4.83	1.74	0.79	5.06
10	0.70	1.22	2.93	10.76	0.40	1.13	3.07
15	1.44	1.48	2.16	8.30	1.04	4.01	5.37

B) Polβ in the presence of XRCC1

Time [min]	dU(0)	dU(-5)	dU(-3)	dU(+/-)3	dU(+/-)5	dU(+)3	dU(+)5
**Experiment 1**	**Percentage of Digestion [%]**						
0	0.00	0.00	0.00	0.00	0.00	0.00	0.00
1	86.26	80.71	61.68	22.24	71.52	63.57	72.51
5	98.69	94.51	87.12	76.81	95.66	74.44	91.28
15	96.03	99.35	98.64	93.75	99.81	68.54	94.83
30	97.59	100.00	96.79	97.31	100.54	76.28	94.62
**Experiment 2**	**Percentage of Digestion [%]**						
0	0.00	0.00	0.00	0.00	0.00	0.00	0.00
1	83.81	80.93	72.15	54.81	75.39	62.20	75.87
5	93.10	93.20	89.20	84.42	92.76	75.72	89.56
15	94.08	97.29	93.95	91.66	95.94	82.53	91.39
30	94.02	97.98	94.43	94.13	97.10	81.79	92.20
**Experiment 3**	**Percentage of Digestion [%]**						
0	0.00	0.00	0.00	0.00	0.00	0.00	0.00
1	90.01	92.88	92.06	90.80	84.42	62.56	82.86
5	93.87	95.34	93.65	94.86	95.49	89.92	88.12
15	94.90	94.69	94.80	92.27	95.76	88.68	88.02
30	94.07	96.59	94.92	93.02	94.36	83.15	87.48
**Time [min]**	**Average Percentage of Digestion [%]**						
0	0.00	0.00	0.00	0.00	0.00	0.00	0.00
1	86.69	84.84	75.30	55.95	77.11	62.78	77.08
5	95.22	94.35	89.99	85.36	94.64	80.03	89.65
15	95.00	97.11	95.80	92.56	97.17	79.92	91.41
30	95.22	98.22	95.38	94.82	97.34	80.41	91.43
	**Standard Deviation**						
0	0.00	0.00	0.00	0.00	0.00	0.00	0.00
1	3.10	6.09	10.70	20.46	4.80	0.29	3.74
5	1.07	1.07	2.38	5.77	1.40	7.28	0.86
15	0.51	1.45	0.93	0.46	0.77	4.50	1.95
30	0.68	0.88	0.47	0.91	1.65	1.37	2.54

C) Polβ in the presence of PCNA

Time [min]	dU(0)	dU(-5)	dU(-3)	dU(+/-)3	dU(+/-)5	dU(+3)	dU(+)5
**Experiment 1**	**Percentage of Digestion [%]**						
0	0	0	0	0	0	0	0
1	84.91	78.90	69.85	45.30	79.49	59.14	76.27
5	94.33	93.93	87.47	85.53	95.9	73.76	92.65
10	94.72	96.85	92.43	95.15	96.63	81.77	92.99
15	96.69	98.04	93.50	96.61	97.77	87.16	95.48
**Experiment 2**	**Percentage of Digestion [%]**						
0	0	0	0	0	0	0	0
1	85.01	79.47	71.03	41.41	81.02	58.31	77.95
5	96.40	94.36	87.82	85.52	97.12	76.80	93.45
10	97.91	96.97	91.66	96.28	97.26	85.95	96.49
15	97.52	98.90	94.27	97.84	99.70	88.67	
**Experiment 3**	**Percentage of Digestion [%]**						
0	0	0	0	0	0	0	0
1	86.84	79.92	73.76	47.46	80.13	55.18	78.41
5	96.44	93.73	88.52	85.83	96.07	74.15	92.36
10	98.21	96.92	92.72	96.12	96.38	80.43	95.49
15	97.65	98.75	91.50	97.02	98.16	86.81	95.28
**Time [min]**	**Average Percentage of Digestion [%]**						
0	0.00	0.00	0.00	0.00	0.00	0.00	0.00
1	85.59	79.43	71.54	44.72	80.21	57.54	77.54
5	95.72	94.00	87.94	85.63	96.40	74.90	92.82
10	96.94	96.91	92.27	95.85	96.75	82.72	94.99
15	97.29	98.56	93.09	97.16	98.55	87.55	98.20
	**Standard Deviation**						
0	0.00	0.00	0.00	0.00	0.00	0.00	0.00
1	1.09	0.51	2.00	3.07	0.77	2.09	1.13
5	1.20	0.31	0.53	0.18	0.63	1.65	0.57
10	1.93	0.06	0.55	0.62	0.46	2.88	1.80
15	0.52	0.46	1.43	0.63	1.02	0.99	4.89

D) nuclear extract of xrs5 cells

Time [min]	dU0	dU(-5)	dU(-3)	dU(+/-)3	dU(+/-)5	dU(+3)	dU(+5)
**Experiment 1**	**Percentage of Digestion [%]**						
0	0.00	0.00	0.00	0	0	0	0
5	27.05	29.60	21.56	0.00	0.00	11.99	0
30	49.79	53.14	51.09	0.00	0.11	44.95	21.23
60	60.45	63.61	64.40	0.00	7.16	60.99	40.42
120	72.59	78.06	72.85	0.91	14.75	73.06	60.11
**Experiment 2**	**Percentage of Digestion [%]**						
0	0.00	0.00	0.00	0.00	0.00	0.00	0.00
5	18.43	53.57	19.85	5.44	8.69	18.46	12.98
30	28.60	67.33	40.42	11.48	16.12	34.85	28.78
60	55.93	83.72	59.46	12.79	29.32	52.85	35.27
120	78.51	90.30	71.78	17.11	34.75	65.49	41.66
**Experiment 3**	**Percentage of Digestion [%]**						
0	0.00	0.00	0.00	0.00	0.00	0.00	0.00
5	25.37	24.71	31.13	5.42	7.23	25.53	13.42
30	43.54	49.01	48.35	10.11	14.02	46.24	33.23
60	58.38	60.40	62.72	11.22	24.87	57.91	37.35
120	69.06	70.98	70.69	13.96	33.15	67.25	58.81
**Time [min]**	**Average Percentage of Digestion [%]**						
0	0.00	0.00	0.00	0.00	0.00	0.00	0.00
5	23.62	35.96	24.18	2.72	3.74	18.66	8.80
30	40.64	56.50	46.62	6.23	10.09	42.01	27.75
60	58.25	69.24	62.19	7.63	20.45	57.25	37.68
120	73.39	79.78	71.77	10.66	27.55	68.60	53.52
	**Standard Deviation**						
0	0.00	0.00	0.00	0.00	0.00	0.00	0.00
5	4.57	15.45	6.08	4.69	7.34	6.77	7.63
30	10.89	9.61	5.54	7.94	8.70	6.24	6.06
60	2.27	12.64	2.51	7.62	11.72	4.11	2.59
120	4.77	9.77	1.08	8.59	11.11	3.96	10.30

E) nuclear extract of xrs5 cells in the PCNA presence of external source

Time [min]	dU0	dU(-5)	dU(-3)	dU(+/-)3	dU(+/-)5	dU(+3)	dU(+5)
**Experiment 1**	**Percentage of Digestion [%]**						
0	0.00	0.00	0.00	0.00	0.00	0.00	0.00
5	13.42	10.16	22.24	0.00	7.97	10.94	1.80
30	38.47	37.32	45.32	0.00	20.44	29.86	23.41
60	53.95	50.09	57.06	4.20	28.14	38.39	29.11
120	59.38	64.36	61.63	14.04	31.36	50.54	35.00
**Experiment 2**	**Percentage of Digestion [%]**						
0	0.00	0.00	0.00	0.00	0.00	0.00	0.00
5	8.28	8.43	8.21	2.17	1.41	9.69	13.25
30	20.86	31.91	23.55	3.33	1.67	20.21	12.88
60	35.14	54.94	39.43	4.39	6.85	40.18	18.97
120	52.80	71.45	54.22	8.66	11.11	61.93	29.00
**Experiment 3**	**Percentage of Digestion [%]**						
0	0.00	0.00	0.00	0.00	0.00	0.00	0.00
5	13.71	17.09	22.35	0.00	1.13	7.61	0.00
30	39.35	40.34	49.90	0.00	18.40	18.71	14.18
60	55.65	48.00	55.96	9.50	25.87	38.93	18.76
120	59.88	61.09	56.45	14.40	26.70	41.19	19.97
**Time [min]**	**Average Percentage of Digestion [%]**						
0	0.00	0.00	0.00	0.00	0.00	0.00	0.00
5	11.80	11.89	17.60	0.00	3.50	9.41	3.10
30	32.89	36.52	39.59	0.00	13.50	22.93	16.82
60	48.25	51.01	50.82	6.03	20.29	39.17	22.28
120	57.35	65.64	57.44	12.37	23.05	51.22	27.99
	**Standard Deviation**						
0	0.00	0.00	0.00	0.00	0.00	0.00	0.00
5	3.06	4.58	8.13	2.73	3.87	1.68	9.56
30	10.43	4.27	14.07	6.75	10.30	6.05	5.74
60	11.38	3.56	9.88	3.01	11.69	0.92	5.92
120	3.95	5.30	3.80	3.22	10.61	10.39	7.57

F) nuclear extract of xrs5 cells in the XRCC1 presence of external source

Time [min]	dU0	dU(-5)	dU(-3)	dU(+/-)3	dU(+/-)5	dU(+3)	dU(+5)
**Experiment 1**	**Percentage of Digestion [%]**						
0	0.00	0.00	0.00	0.00	0.00	0.00	0.00
5	10.23	6.27	12.36	0.00	0.79	13.72	6.06
30	34.83	36.63	40.15	0.27	10.40	32.74	24.46
60	50.89	49.09	54.71	3.39	21.96	44.25	34.41
120	65.11	62.39	64.75	3.84	29.13	52.48	44.20
**Experiment 2**	**Percentage of Digestion [%]**						
0	0.00	0.00	0.00	0.00	0.00	0.00	0.00
5	30.03	21.29	31.50	6.56	11.13	18.80	17.69
30	46.99	38.92	50.80	8.70	24.21	33.53	29.29
60	57.92	48.80	60.35	12.36	36.77	38.17	35.19
120	66.92	60.27	64.22	14.63	47.14	52.66	46.93
**Experiment 3**	**Percentage of Digestion [%]**						
0	0.00	0.00	0.00	0.00	0.00	0.00	0.00
5	6.45	7.49	8.01	4.42	6.97	9.86	10.29
30	28.55	37.79	30.26	5.66	13.20	36.48	17.32
60	43.81	61.86	47.95	5.93	15.80	51.61	29.15
120	61.51	77.49	64.20	11.72	22.79	66.49	44.16
**Time [min]**	**Average Percentage of Digestion [%]**						
0	0.00	0.00	0.00	0.00	0.00	0.00	0.00
5	15.57	11.68	17.29	3.61	6.30	14.12	11.35
30	36.79	37.78	40.40	4.88	15.94	34.25	23.69
60	50.88	53.25	54.34	7.23	24.84	44.68	32.92
120	64.51	66.72	64.39	10.06	33.02	57.21	45.09
	**Standard Deviation**						
0	0.00	0.00	0.00	0.00	0.00	0.00	0.00
5	12.66	8.34	12.50	3.43	5.20	4.48	5.88
30	9.38	1.14	10.27	4.27	7.30	1.97	6.02
60	7.05	7.46	6.21	4.62	10.78	6.73	3.28
120	2.76	9.39	0.31	5.58	12.63	8.04	1.59

G) nuclear extract of xrs5 cells in the Polβ presence of external source

Time [min]	dU0	dU(-5)	dU(-3)	dU(+/-)3	dU(+/-)5	dU(+3)	dU(+5)
**Experiment 1**	**Percentage of Digestion [%]**						
0	0.00	0.00	0.00	0.00	0.00	0.00	0.00
5	90.23	90.82	90.59	85.99	91.99	77.88	92.45
30	97.58	100.00	99.12	99.82	98.37	87.39	95.08
60	99.00	100.00	100.00	100.00	99.74	93.36	95.85
120	99.51	100.00	100.00	100.00	100.00	94.09	95.93
**Experiment 2**	**Percentage of Digestion [%]**						
0	0.00	0.00	0.00	0.00	0.00	0.00	0.00
5	91.72	96.65	86.31	92.49	90.94	74.98	89.89
30	97.68	100.00	98.25	98.43	98.50	83.78	93.73
60	99.11	100.00	98.84	98.90	99.86	85.58	94.79
120	99.28	100.00	96.09	99.26	99.98	90.25	95.92
**Experiment 3**	**Percentage of Digestion [%]**						
0	0.00	0.00	0.00	0.00	0.00	0.00	0.00
15	91.61	95.08	92.44	90.13	93.09	51.07	92.03
30	96.11	99.06	96.90	97.05	97.92	36.43	90.49
60	97.89	99.31	97.77	97.84	99.08	64.40	91.49
120	98.72	99.61	97.81	98.51	99.49	82.60	93.76
**Time [min]**	**Average Percentage of Digestion [%]**						
0	0.00	0.00	0.00	0.00	0.00	0.00	0.00
15	91.19	94.19	89.78	89.54	92.01	67.98	91.46
30	97.12	99.81	98.09	98.43	98.26	69.20	93.10
60	98.67	100.00	98.87	99.03	99.56	81.11	94.04
120	99.17	100.00	98.02	99.35	100.00	88.98	95.20
	**Standard Deviation**						
0	0.00	0.00	0.00	0.00	0.00	0.00	0.00
15	0.83	3.01	3.15	3.29	1.08	14.71	1.38
30	0.88	0.67	1.12	1.39	0.31	28.44	2.36
60	0.68	0.76	1.12	1.27	0.42	14.99	2.28
120	0.41	0.55	2.04	0.90	0.57	5.85	1.25

The repair assay of oligonucleotides after UDG and HAPE1 digestion, by:

H) nuclear extract of xrs5 cells

Time [min]	dU0	dU(-5)	dU(-3)	dU(+/-)3	dU(+/-)5	dU(+3)	dU(+5)
**Experiment 1**	**Percentage of Digestion [%]**						
0	0.00	0.00	0.00	0.00	0.00	0.00	0.00
5	2.20	1.46	2.18	1.81	0.00	0.00	0.00
30	12.75	1.55	16.85	1.76	0.00	0.00	0.00
60	28.02	3.24	25.67	1.91	0.00	0.00	0.00
120	38.57	6.48	34.16	1.18	0.00	0.00	0.00
**Experiment 2**	**Percentage of Digestion [%]**						
0	0.00	0.00	0.00	0.00	0.00	0.00	0.00
5	4.30	1.45	4.16	2.38	0.00	0.00	0.00
30	10.59	2.22	11.54	1.63	0.00	0.00	0.00
60	18.67	3.26	20.78	2.11	0.00	0.00	0.00
120	21.74	4.35	31.38	2.28	0.00	0.00	0.00
**Experiment 3**	**Percentage of Digestion [%]**						
0	0.00	0.00	0.00	0.00	0.00	0.00	0.00
5	1.49	1.34	1.77	1.18	0.00	0.00	0.00
30	7.63	1.39	14.78	1.14	0.00	0.00	0.00
60	25.34	3.16	28.43	1.51	0.00	0.00	0.00
120	35.66	5.75	34.64	0.83	0.00	0.00	0.00
**Time [min]**	**Average Percentage of Digestion [%]**						
0	0.00	0.00	0.00	0.00	0.00	0.00	0.00
5	0.00	1.42	2.70	0.00	0.00	0.00	0.00
30	10.32	1.72	14.39	0.00	0.00	0.00	0.00
60	24.01	3.22	24.96	0.00	0.00	0.00	0.00
120	31.99	5.53	33.39	0.00	0.00	0.00	0.00
	**Standard Deviation**						
0	0.00	0.00	0.00	0.00	0.00	0.00	0.00
5	1.46	0.06	1.28	0.00	0.00	0.00	0.00
30	2.57	0.44	2.68	0.00	0.00	0.00	0.00
60	4.82	0.05	3.87	0.00	0.00	0.00	0.00
120	9.00	1.08	1.76	0.00	0.00	0.00	0.00

I) nuclear extract of xrs5 cells in the PCNA presence of external source.

Time [min]	dU0	dU(-5)	dU(-3)	dU(+/-)3	dU(+/-)5	dU(+3)	dU(+5)
**Experiment 1**	**Percentage of Digestion [%]**						
0	0.00	0.00	0.00	0.00	0.00	0.00	0.00
5	0.00	0.19	0.00	0.00	0.00	0.00	0.00
30	6.11	0.00	10.48	0.00	0.00	0.00	0.00
60	20.54	2.29	25.34	0.00	0.00	0.00	0.00
120	30.22	11.07	31.95	0.00	0.00	0.00	0.00
**Experiment 2**	**Percentage of Digestion [%]**						
0	0.00	0.00	0.00	0.00	0.00	0.00	0.00
5	1.49	1.08	1.15	0.00	0.00	0.00	0.00
30	5.54	2.37	5.63	0.00	0.00	0.00	0.00
60	13.26	3.60	14.70	0.00	0.00	0.00	0.00
120	24.93	5.71	19.43	0.00	0.00	0.00	0.00
**Experiment 3**	**Percentage of Digestion [%]**						
0	0.00	0.00	0.00	0.00	0.00	0.00	0.00
5	-4.48	0.64	0.26	0.00	0.00	0.00	0.00
30	11.76	0.49	19.34	0.00	0.00	0.00	0.00
60	24.10	3.19	24.37	0.00	0.00	0.00	0.00
120	30.48	9.25	25.85	0.00	0.00	0.00	0.00
**Time [min]**	**Average Percentage of Digestion [%]**						
0	0.00	0.00	0.00	0.00	0.00	0.00	0.00
5	0.00	0.64	0.47	0.00	0.00	0.00	0.00
30	7.80	0.95	11.82	0.00	0.00	0.00	0.00
60	19.30	3.03	21.47	0.00	0.00	0.00	0.00
120	28.55	8.68	25.74	0.00	0.00	0.00	0.00
	**Standard Deviation**						
0	0.00	0.00	0.00	0.00	0.00	0.00	0.00
5	3.11	0.45	0.60	0.00	0.00	0.00	0.00
30	3.44	1.25	6.95	0.00	0.00	0.00	0.00
60	5.53	0.67	5.89	0.00	0.00	0.00	0.00
120	3.13	2.73	6.26	0.00	0.00	0.00	0.00

J) nuclear extract of xrs5 cells in the XRCC1 presence of external source

Time [min]	dU0	dU(-5)	dU(-3)	dU(+/-)3	dU(+/-)5	dU(+3)	dU(+5)
**Experiment 1**	**Percentage of Digestion [%]**						
0	0.00	0.00	0.00	0.00	0.00	0.00	0.00
5	0.92	0.00	0.00	0.00	0.00	0.00	0.00
30	7.11	0.00	10.90	0.00	0.00	0.00	0.00
60	22.15	0.37	25.39	0.00	0.00	0.00	0.00
120	37.83	3.03	33.88	0.00	0.00	0.00	0.00
**Experiment 2**	**Percentage of Digestion [%]**						
0	0.00	0.00	0.00	0.00	0.00	0.00	0.00
5	2.40	0.88	1.67	0.00	0.00	0.00	0.00
30	14.94	1.47	18.14	0.00	0.00	0.00	0.00
60	28.08	3.91	29.09	0.00	0.00	0.00	0.00
120	40.84	5.77	35.88	0.00	0.00	0.00	0.00
**Experiment 3**	**Percentage of Digestion [%]**						
0	0.00	0.00	0.00	0.00	0.00	0.00	0.00
5	0.00	0.00	0.00	0.00	0.00	0.00	0.00
30	6.66	0.75	6.23	0.00	0.00	0.00	0.00
60	16.02	3.69	14.75	0.00	0.00	0.00	0.00
120	28.61	5.40	22.49	0.00	0.00	0.00	0.00
**Time [min]**	**Average Percentage of Digestion [%]**						
0	0.00	0.00	0.00	0.00	0.00	0.00	0.00
5	0.99	0.00	0.28	0.00	0.00	0.00	0.00
30	9.57	0.74	11.76	0.00	0.00	0.00	0.00
60	22.08	2.66	23.08	0.00	0.00	0.00	0.00
120	35.76	4.73	30.75	0.00	0.00	0.00	0.00
	**Standard Deviation**						
0	0.00	0.00	0.00	0.00	0.00	0.00	0.00
5	1.38	0.90	1.27	0.00	0.00	0.00	0.00
30	4.65	0.73	6.00	0.00	0.00	0.00	0.00
60	6.03	1.98	7.44	0.00	0.00	0.00	0.00
120	6.38	1.49	7.22	0.00	0.00	0.00	0.00

K) nuclear extract of xrs5 cells in the Polβ presence of external source

Time [min]	dU0	dU(-5)	dU(-3)	dU(+/-)3	dU(+/-)5	dU(+3)	dU(+5)
**Experiment 1**	**Percentage of Digestion [%]**						
0	0.00	0.00	0.00	0.00	0.00	0.00	0.00
15	1.56	0.00	4.73	0.00	0.00	2.40	0.90
30	4.03	1.72	8.20	1.34	1.36	2.22	1.43
60	5.90	4.11	13.88	3.52	3.90	2.85	3.26
120	11.89	12.01	26.80	11.94	25.83	7.24	11.49
**Experiment 2**	**Percentage of Digestion [%]**						
0	0.00	0.00	0.00	0.00	0.00	0.00	0.00
15	0.92	0.90	2.73	0.88	1.08	0.68	-0.35
30	2.75	0.78	3.65	1.55	1.28	1.13	0.15
60	10.68	2.25	7.17	3.64	2.44	2.06	1.03
120	32.45	3.62	18.24	6.64	7.70	4.82	11.55
**Experiment 3**	**Percentage of Digestion [%]**						
0	0.00	0.00	0.00	0.00	0.00	0.00	0.00
15	3.19	0.00	2.26	0.00	0.00	0.00	0.00
30	6.19	0.13	6.43	0.91	0.09	1.08	0.22
60	12.21	0.94	10.30	2.99	1.81	5.08	4.27
120	19.59	5.56	27.01	18.08	8.66	16.65	24.76
**Time [min]**	**Average Percentage of Digestion [%]**						
0	0.00	0.00	0.00	0.00	0.00	0.00	0.00
15	1.89	0.00	3.24	0.00	0.02	0.89	0.00
30	4.32	0.88	6.09	1.27	0.91	1.47	0.60
60	9.60	2.43	10.45	3.38	2.71	3.33	2.85
120	21.31	7.07	24.02	12.22	14.06	9.57	15.93
	**Standard Deviation**						
0	0.00	0.00	0.00	0.00	0.00	0.00	0.00
15	1.17	1.05	1.31	1.41	1.00	1.42	1.06
30	1.74	0.80	2.30	0.33	0.71	0.65	0.72
60	3.29	1.59	3.36	0.35	1.07	1.57	1.66
120	10.39	4.39	5.01	5.72	10.20	6.25	7.64

Spectra of Oligonucleotide Mass Spectrometry Analysis

Matrix (mass calculated: 12409.14)

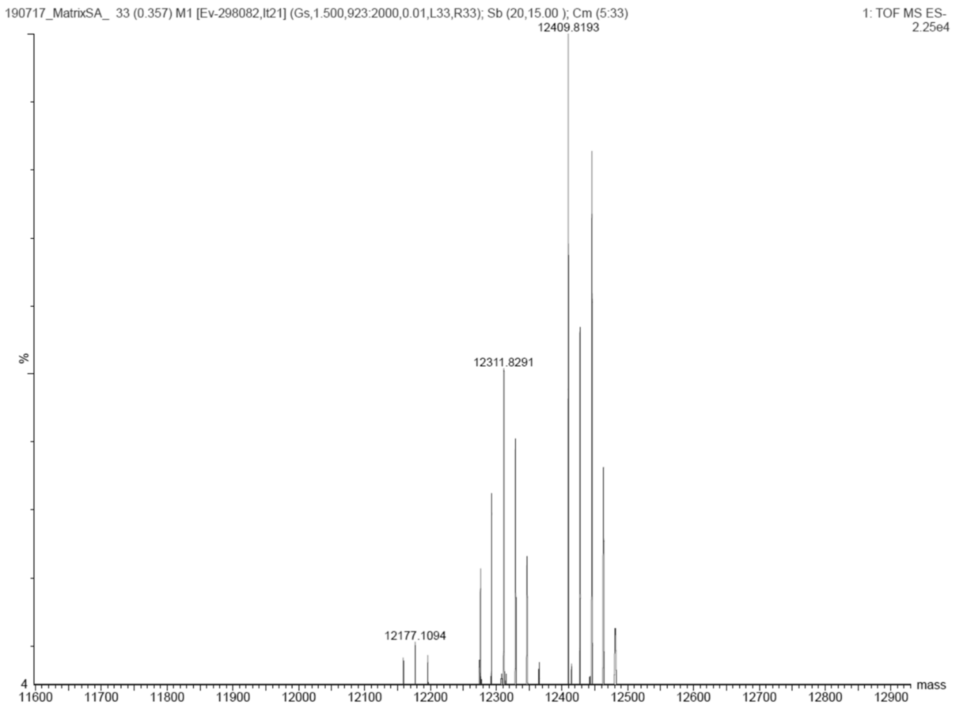

Cont.dU(0) (mass calculated: 12167.90)

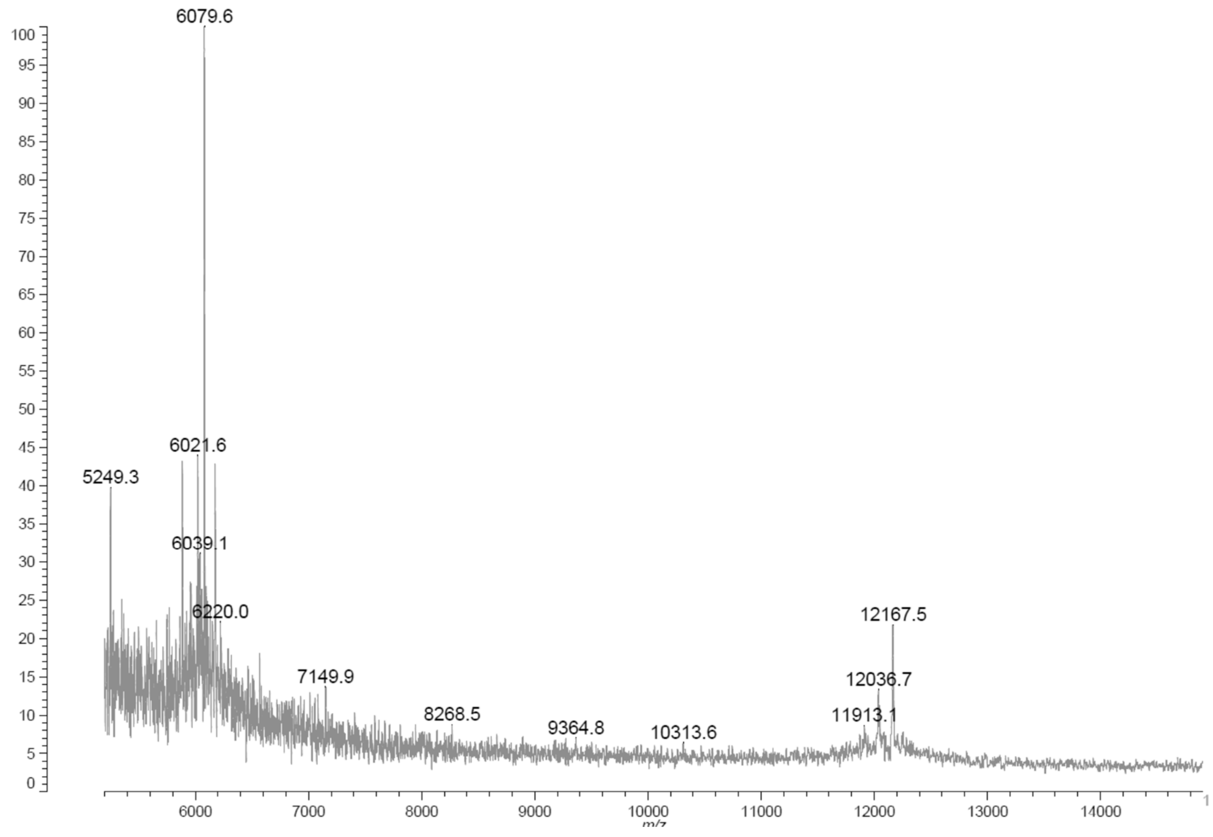

dU(-5) (mass calculated: 12165.90)

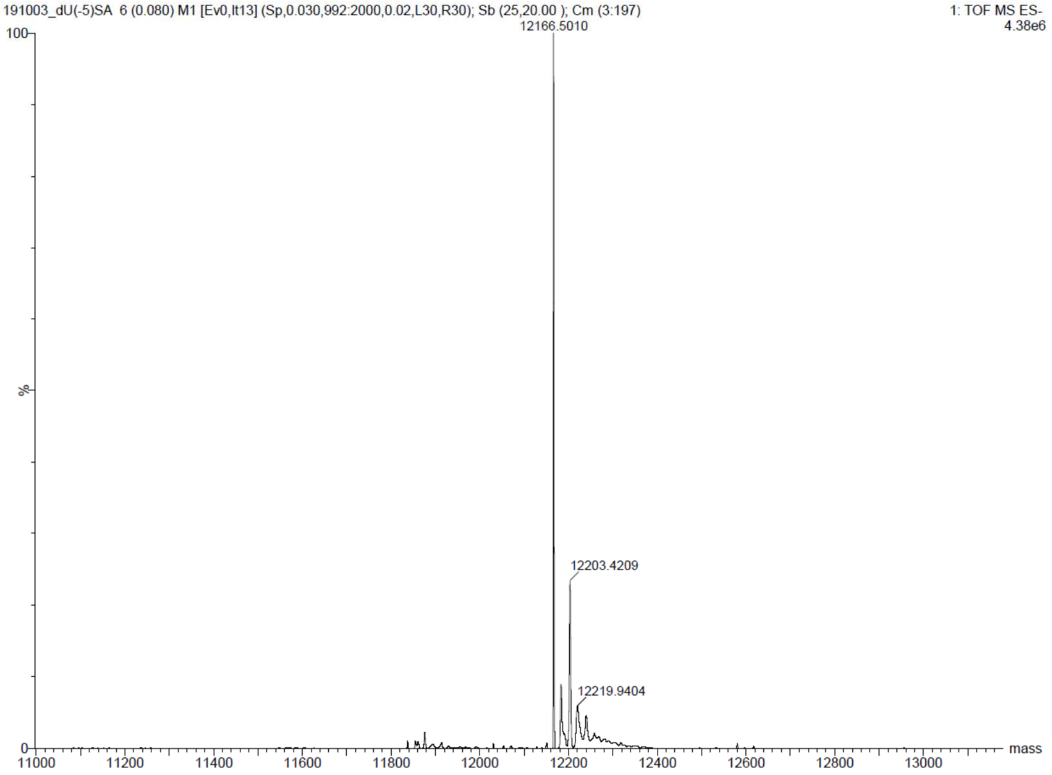

dU(-3) (mass calculated: 12165.90)

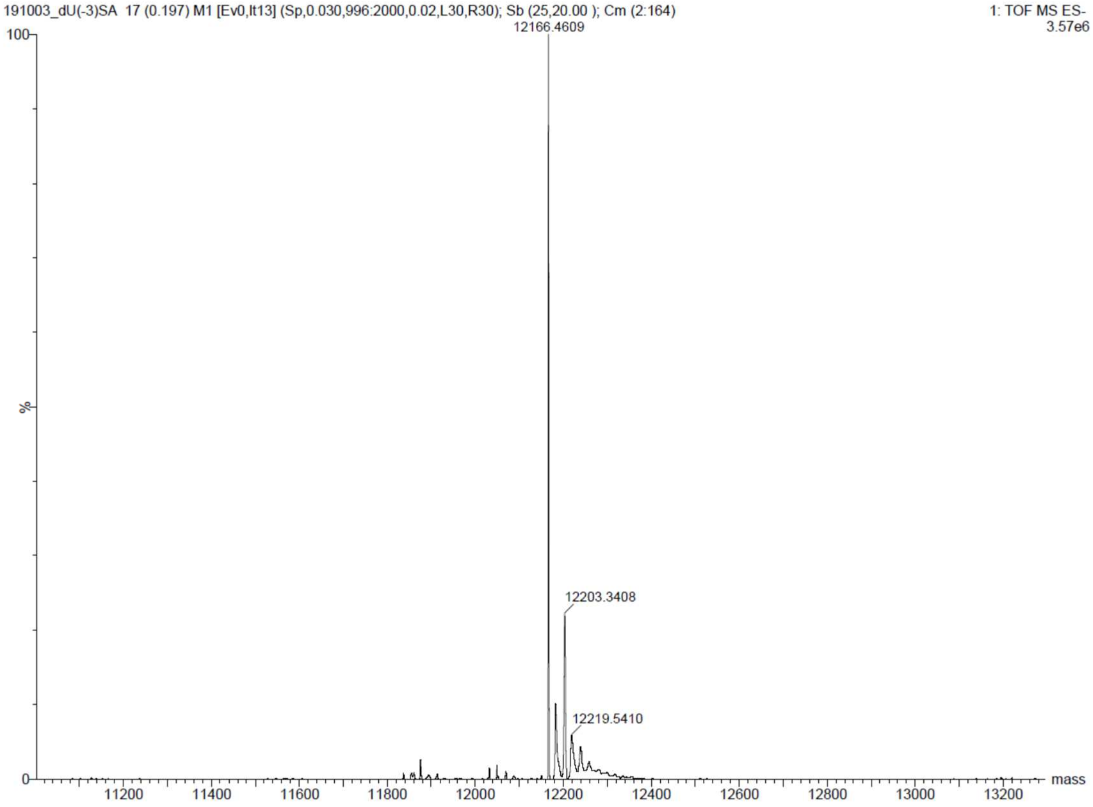

dU(+3) (mass calculated: 12180.90)

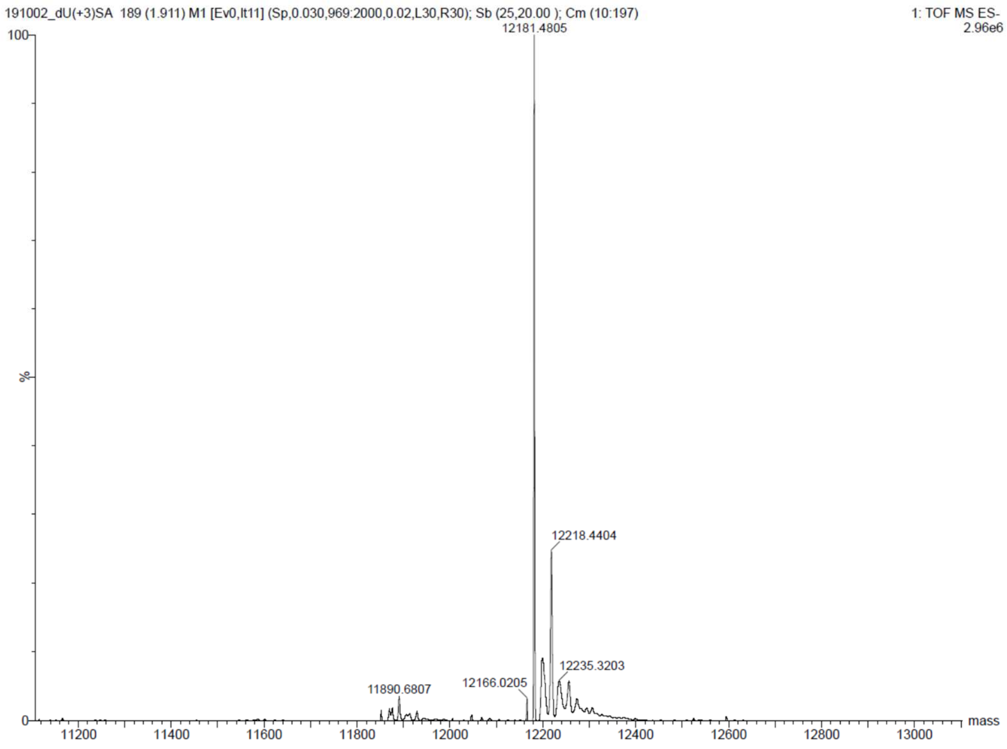

dU(+5) (mass calculated: 12180.90)

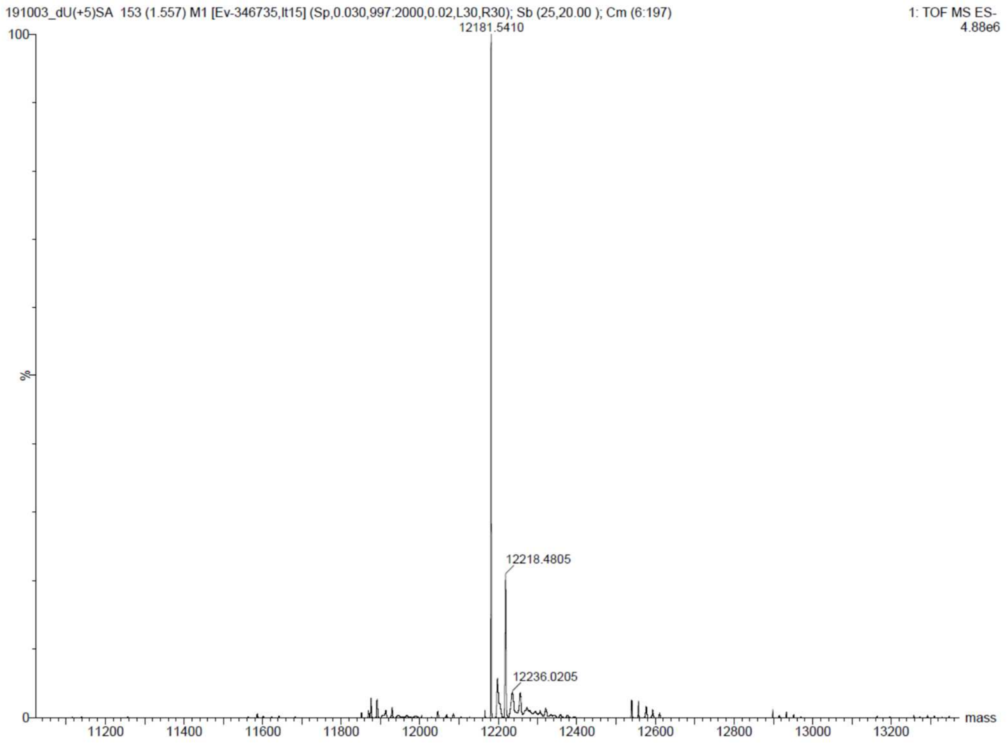

dU(+/-)3 (mass calculated: 12166.89)

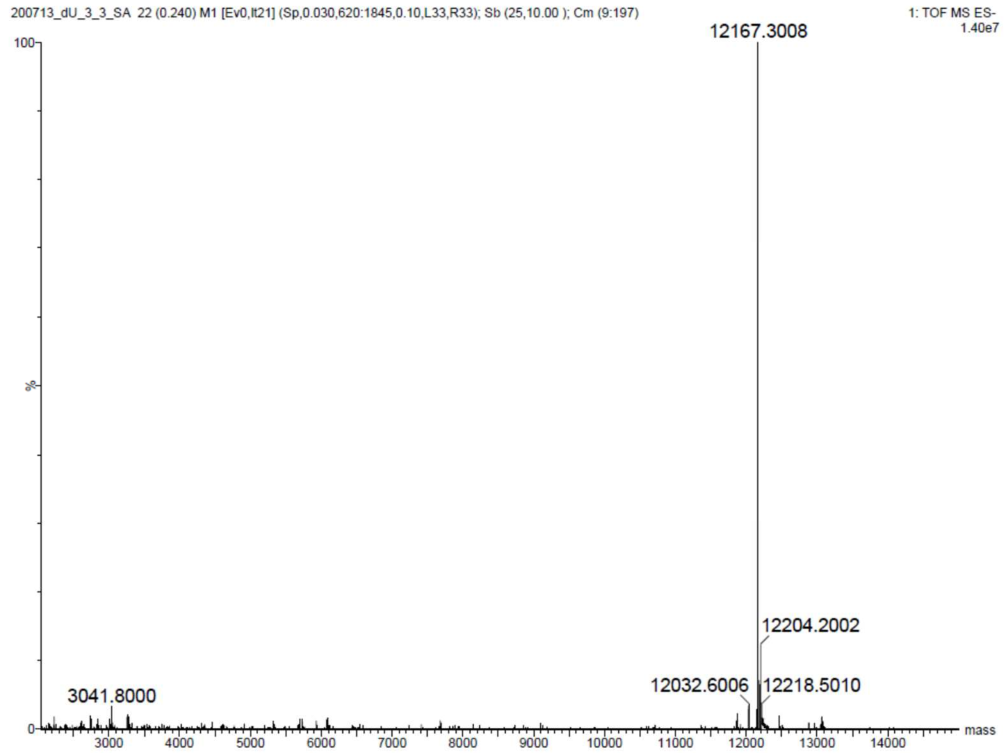

dU(+/-)5 (mass calculated: 12166.89)

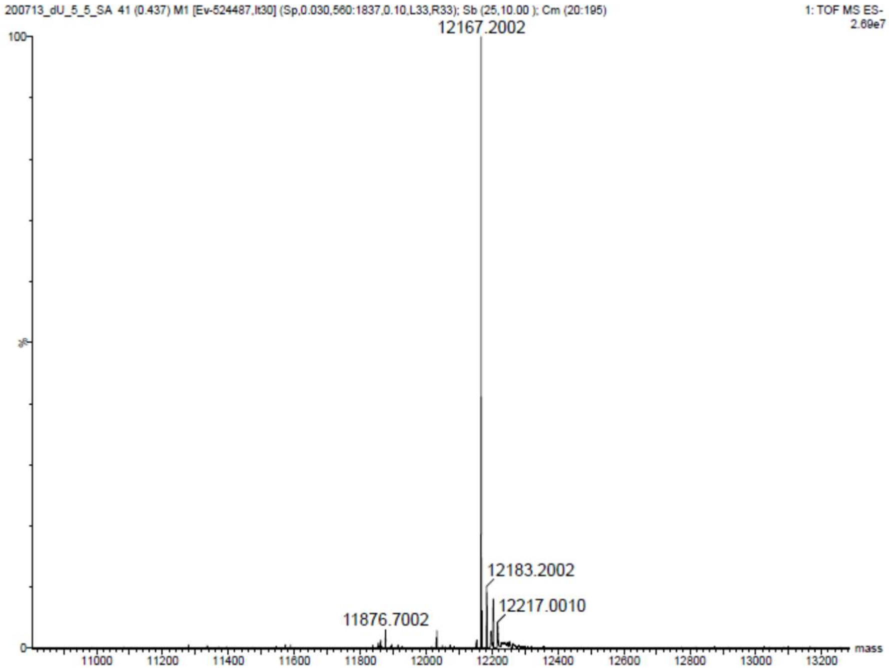

Cont.cdA (mass calculated: 12407.16)

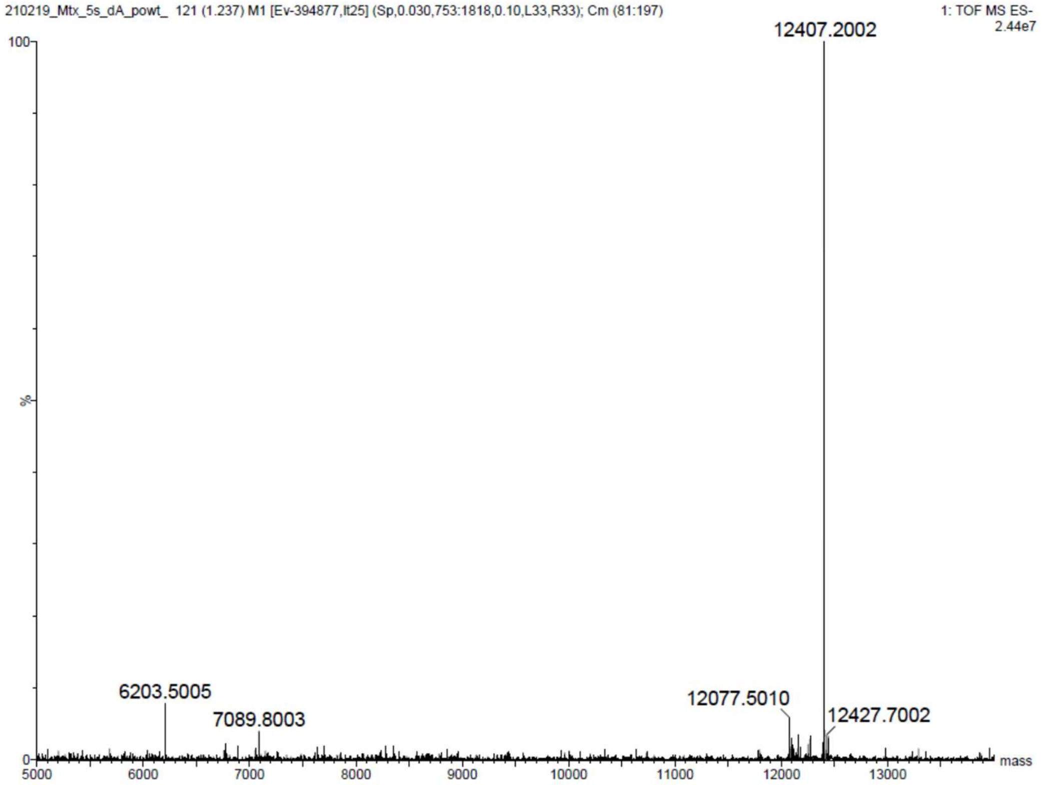

Supplement: Supplementary file 1 [file ijms-22-05934-s001.zip › ijms-1221157-supplementary.pdf]
